# Identification of Three Novel Tetrahydrocannabinol Analogs in the European Market

**DOI:** 10.1002/dta.3866

**Published:** 2025-02-06

**Authors:** Evangelos Dadiotis, Sotiris Mpakaoukas, Vangelis Mitsis, Eleni Melliou, Prokopios Magiatis

**Affiliations:** ^1^ Laboratory of Pharmacognosy and Natural Products Chemistry, Department of Pharmacy National and Kapodistrian University of Athens Athens Greece; ^2^ Ekati Alchemy Lab SL Barcelona Spain

**Keywords:** Cannabis public health, CB9, CBx, semisynthetic cannabinoids, tresconol

## Abstract

Synthetic cannabinoids, known as Spice or K2, emerged in Europe and the United States between 2005 and 2008, peaking in incidents by 2015 with severe health implications. In 2021, the identification of hexahydrocannabinol (HHC), a semisynthetic cannabinoid (SSC), led to regulatory control in more than 20 countries in Europe, several US states, and other jurisdictions. A 2024 study in the United States highlighted the diversity of semisynthetic cannabinoids in the US market. New entries of SSCs are increasingly available in the European market, often found as blends in consumer products. This highlights the growing complexity of their regulation and the potential public health risks due to limited toxicological data. The major ingredients, isolated from “CB9”, “tresconol”, and “CBx”, were subjected to mass spectrometry (MS) and nuclear magnetic resonance (NMR) spectroscopy. The major isolated ingredient of “CB9” was identified as [2‐(*E*)‐propen‐1‐yl]‐Δ^8^‐tetrahydrocannabinol‐acetate. The major ingredient of “tresconol” was identified as [2‐propen‐2‐yl]‐Δ^9^‐tetrahydrocannabinol, and the major ingredient of “CBx” was identified as [2‐propen‐2‐yl]‐Δ^8^‐tetrahydrocannabinol. These compounds have no available spectroscopic or chromatographic data, have never been identified in *Cannabis* plants, and cannot be identified by standard chromatographic forensic analytical methods, without the use of spectroscopic techniques due to the lack of reference standards. The products were complex mixtures of previously unknown synthetic cannabinoids lacking established safety profiles. These findings highlight the potential public health risks associated with unregulated SSCs, similar to the concerns raised during the 2015 Spice outbreak. The presence of these novel substances requires careful monitoring to prevent future health crises.

## Graphical abstract

The major isolated ingredient of “CB9” was identified as [2‐(*E*)‐propen‐1‐yl]‐Δ^8^‐tetrahydrocannabinol‐acetate of “tresconol” as [2‐propen‐2‐yl]‐Δ^9^‐tetrahydrocannabinol and of “CBx” as [2‐propen‐2‐yl]‐Δ^8^‐tetrahydrocannabinol. These compounds have no available spectroscopic or chromatographic data and have never been identified in *Cannabis* plants, and their presence requires careful monitoring to prevent future health crises.

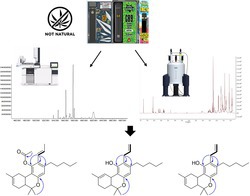

## 1 Introduction

Synthetic cannabinoids, also known as Spice or K2, first appeared in Europe in 2005 and in the United States in 2008. The peak of the incidents occurred in 2015 when poison centers in the United States received 7794 calls about poisoning from synthetic cannabinoids [1, 2]. The consumption of Spice in the past has been associated with various adverse effects, including but not limited to acute kidney injury, seizures, psychosis, cardiotoxic effects, coma, and death [3]. Currently, a variety of synthetic and semisynthetic cannabinoids (SSC), also known as tetrahydrocannabinol (THC) analogs, is emerging. According to the European Union Drugs Agency (EUDA), an SSC named hexahydrocannabinol (HHC) was reported for the first time in the US market in September 2021 and in the European market in May 2022 [4]. Although HHC was first described in scientific literature back in 1940 [5], little is known about its health risks, with more information about HHC available in Ujvary's review [6]. In 2023, two more derivatives of HHC were identified in the US market: HHC acetate (HHCO) and hexahydrocannabiphorol (HHCP). Products containing HHC and its derivatives, such as vape pens, e‐liquids, e‐liquid cartridges for e‐cigarettes, edibles, oils, and sprayed hemp plants, are available in online and physical stores [4]. Moreover, products with semisynthetic cannabinoids seem to have discrepancies between the stated contents and analytical findings, containing novel SSC [7]. More than 20 European countries have adopted legislative controls, with the Czech Republic being the latest to control HHC after the hospitalization of 12 children, which consumed edibles containing HHC [8]. An online assessment study conducted in the United States during 2023 identified several semisynthetic cannabinoids, such as Δ^8^‐THC, tetrahydrocannabiphorol (THC‐P), Δ^9^‐THC (derived from acidic catalyzed ring closure of CBD), HHC, THC‐A, Δ^10^‐THC, tetrahydrocannbihexol (THC‐H), tetrahydrocannabibutol (THC‐B), THC‐JD, THC‐X, HHC‐P, and Δ^11^‐THC. Most of these products (54%) were blends, containing two to eight different intoxicating compounds in a single product [9]. A recent study in Japan used nuclear magnetic resonance (NMR) and mass spectrometry (MS) to identify HHC, dihydro‐iso‐tetrahydrocannabinol (dihydro‐iso‐THC), and HHCP in electronic cigarette cartridges [10]. In parallel, a review about acid‐catalyzed ring closure of cannabidiol (ACRCC) by‐products associated the use of Δ^8^‐THC products and their byproducts with an increase in electronic cigarette, or vaping, product use–associated lung injury (EVALI) incidents, a serious medical condition evoked by acetate esters of tocopherol acetate or acetate esters of cannabinoids contained in e‐cigarettes [11]. Additionally to Europe, studies have been made also in United States to highlight the existence and distribution of semi synthetic cannabinoids and THC derivatives, especially compounds with the acetate esters of Δ^8^‐THC and Δ^9^‐THC [12].

Currently, the market is introducing various novel semisynthetic and synthetic cannabinoids, including but not limited products available with commercial names “CB9”, “CBx”, and “tresconol” in online and physical shops throughout Europe that are advertised as “natural” and “hemp‐derived.” The aim of the current work was to investigate the chemical consistency of those products and their potential origin. The semisynthetic or synthetic origins and classification of these three new entries as Δ^8^‐THC or Δ^9^‐THC derivatives were confirmed through analyses by NMR and MS techniques.

## 2 Material and Methods

### 2.1 Chemicals

Solvents were procured from commercial sources (Sigma‐Aldrich) and used as received. SSC containing products were provided by the Ekati Alchemy Lab (Spain) and immediately dissolved in Chloroform(−d) for NMR and GC‐MS analysis (acquired as samples from raw cannabinoid European vendors).

### 2.2 NMR Analysis

One‐ and two‐dimensional characterization of NMR experiments was performed on a Bruker Avance III spectrometer equipped with a BBIz 5‐mm probe (400 MHz for ^1^H, and 101 MHz for ^13^C). Spectra were acquired using deuterated chloroform (CDCl_3_). Chemical shifts (δ) for ^1^H‐NMR spectra are reported in parts per million (ppm), using the residual nondeuterated solvent resonance as the internal standard (for CDCl_3_: 7.26 ppm, ^1^H, and 77.16 ppm, ^13^C). Data are reported as follows: chemical shift, multiplicity, coupling constants (*J*) in Hertz (Hz), and integration.

### 2.3 GC‐MS and HRMS Analyses

GC‐MS analysis was performed on an Agilent Technologies 7820A GC System gas chromatograph in combination with the Agilent Technologies 5977B MSD mass spectrometer. The column used was an HP‐5 MS (DB‐5) with dimensions of 30 m × 0.25 mm and a film thickness of 0.25 μm. The ionization mode used was electron ionization (EI) with an ionization energy of 70 eV. Helium (He) was used as the inert gas for the mobile phase, with a flow rate of 1.4 mL/min. The thermal program used for the analysis of the compounds started at a temperature of 60°C with a ramp rate of 3°C/min to 280°C, followed by 17 min to 280°C, with a total run time of 90 min. The inlet temperature was set at 250°C, as was the detector temperature. Samples (1 μL) were injected in split mode with a split ratio of 20:1 and a split flow rate of 18.736 mL/min.

HRMS analysis was performed using a MaXis plus MQ ESI‐QqTOF mass spectrometer (Bruker, Bremen, Germany). The ionization took place in electrospray (two‐bar pressure for nebulizer and 10 L/min for nitrogen dry gas flow) in positive ion mode. End plate offset (500 V) and capillary voltage (4500 V) permitted the ions transfer. To recalibrate spectrum, four times diluted calibrant ESI‐L Low Concentration Tuning Mix (Agilent, Les Ulis, France) was injected at the beginning of each sample. Before batch analysis, the mass spectrometer was calibrated using undiluted Tuning Mix in enhanced quadratic mode (errors < 0.5 ppm). The mass range was between 100 and 1500 *m/z*.

### 2.4 Isolation

Semipreparative high performance liquid chromatography performed on a Jasco HPLC 4000 series system with a semiprep C18 Hedera ODS‐2 (10.0 × 250, 5 μm, 100 Å) column. The mobile phase was water (A) and acetonitrile (B). Elution was performed with an isocratic method of 10% A and 90% B, with 5 mL/min flow rate, and the detector was set at 220 nm.

## 3 Results and Discussion

### 3.1 GC‐MS Analysis

Initially, the commercial cannabinoid containing product “CB9” was analyzed with GC‐MS, and a complex mixture was revealed. From GC‐MS analysis, at least five different compounds were identified. The major peak with [M]^·+^ = 396.3 had 37% of the total peak area (Figure S1). “Tresconol” mixture was also analyzed by GC‐MS, and a major peak with [M]^·+^ = 354.3 was identified with 65% area of the total peak area (Figure S2). Moreover, a major component with [M]^·+^ = 354.3 was detected with GC‐MS analysis of the “CBx” mixture, from a very complex mixture of compounds (Figure S3). In the mass spectrometric analysis of the major compounds identified from the products, “CB9”, “tresconol”, and “CBx” key fragments were observed that provided crucial insights into their structural elucidation. For the major compound of “CB9”, the molecular ion peak at *m/z* 396.3 indicated the intact molecule, whereas significant fragment ions were detected at *m/z* 354, corresponding to a possible loss of a ketene group (C₂H₂O, *m/z* 42) (Figure S1). This loss is characteristic of acetylated cannabinoids and indicates the presence of an acetate group at the C‐1 position. Additionally, for “tresconol” and “CBx” the molecular ion peaks at *m/z* 354.2 were accompanied by fragment ions at *m/z* 339.2, 311.2, 271.1, and 233.1, corresponding to a potential loss of a methyl group and a ring opening, a loss of an allyl group and the pentyl side chain (Figures S2 and S3). The observed fragmentations align well with the known behavior of Δ^9^‐THC and Δ^8^‐THC [13].

### 3.2 Isolation

Each major SSC was isolated from three different commercially available cannabinoid containing products, using semipreparative high‐performance liquid chromatography (HPLC) under an isocratic method with a 90:10 acetonitrile to water ratio. The primary compound from the “CB9” mixture was isolated at a retention time of 5.5 min (Figure S8). Similarly, the major compound from the “tresconol” mixture was collected at a retention time of 4 min (Figure S19), and the major compound from the “CBx” mixture was collected at a retention time of 4.2 min (Figure S30). All the isolated compounds and the original cannabinoid mixtures were subsequently subjected to NMR analysis (Figures S7, S9, S18, S20, S29, and S31).

### 3.3 NMR Analysis

#### 3.3.1 [2‐(*E*)‐Propen‐1‐yl]‐Δ^8^‐Tetrahydrocannabinol‐Acetate

**FIGURE 1:**
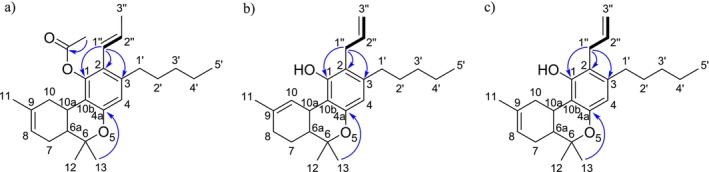
Key HMBC (arrows) and COSY (bold lines) correlations validating the presence of propenyl (a) and allyl (b, c) group in “CB9”, “tresconol”, and “CBx”, respectively.

The ^1^H‐NMR analysis of the major ingredient of CB9 showed a multiplet peak at 5.81 ppm (H‐2″) and a doublet of quartets peak at 6.15 ppm (H‐1″) peak with coupling constants 15.9 and 1.7 Hz, revealing an (*E*) conformation of alkene protons. Additionally, one more singlet at 6.57 ppm (H‐4) and a broad singlet at 6.04 ppm (H‐8) were identified in the aromatic or vinylic region. Through ^1^H‐^13^C HMBC NMR analysis, only one aromatic proton peak at 6.57 ppm was identified revealing a substitution on the aromatic ring of the terpenophenolic cannabinoid skeleton [14]. Neutral cannabinoids have two aromatic protons (H‐2 and H‐4) with a meta‐coupling between them in Δ^8^‐THC spectrum, which in this case was not visible Additionally, a correlation between proton H‐1″ at 6.15 ppm and C‐1 at 147.16 ppm and a correlation between the methyl proton H‐13 at 1.41 ppm and C‐4a at 152.78 ppm revealed that the propenyl group was located at C‐2 (Figure 1). A correlation between the peaks at 5.81 and 6.15 ppm was identified from the ^1^H‐^1^H COSY NMR spectrum. The HMBC NMR analysis also revealed a correlation between the vinylic proton at 5.81 peak and a carbon at 19.39 ppm (C‐3″). From the ^1^H‐^13^C HSQC‐DEPT NMR, a methyl carbon was identified at 19.39 ppm, revealing the presence of a propenyl moiety. A singlet peak at 2.23 (O=C‐CH_3_) peak integrating for three protons and a correlation with an ester carbon at 170.01 ppm from the HMBC analysis revealed the presence of an acetate group (Figures S5–S10). The major compound of the “CB9” product was identified as the [2‐(*E)*‐propen‐1‐yl]‐Δ^8^‐tetrahydrocannabinol‐acetate (IUPAC name: (6a*R*,10a*R*)‐6,6,9‐trimethyl‐3‐pentyl‐2‐((*E*)‐prop‐1‐en‐1‐yl)‐6a,7,10,10a‐tetrahydro‐6*H*‐benzo[*c*]chromen‐1‐yl acetate) (Table 1) (Figures S9–S13). From the ^1^H‐NMR analysis, [2‐(*Z*)‐propen‐1‐yl]‐Δ^8^‐tetrahydrocannabinol‐acetate (“*cis* isomer”) was also identified as a minor ingredient of the mixture (Figure S7). A *Reaxys* literature search resulted in no result for [2‐(*E*)‐propen‐1‐yl]‐Δ^8^‐tetrahydrocannabinol‐acetate, indicating that is a novel SSC, previously unidentified in both literature and the *Cannabis* plant.

**TABLE 1:** New entries of semisynthetic cannabinoids in the European market.

Semisynthetic cannabinoids (SSCs)^a^						
	Commercial name/structure/chemical name/molecular weight					
	CB9 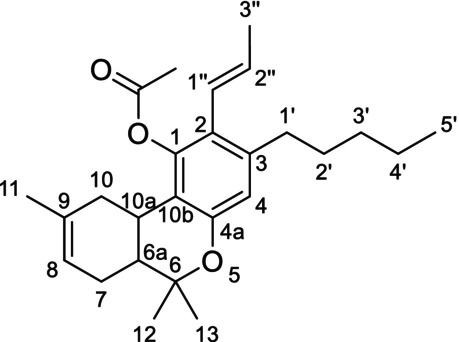		Tresconol 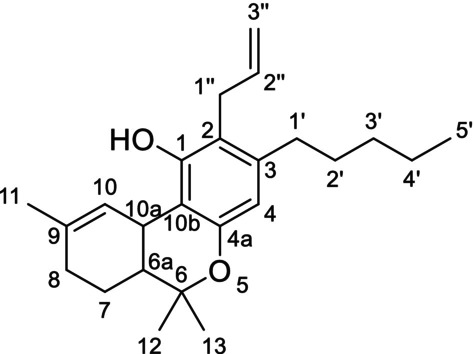		CBx 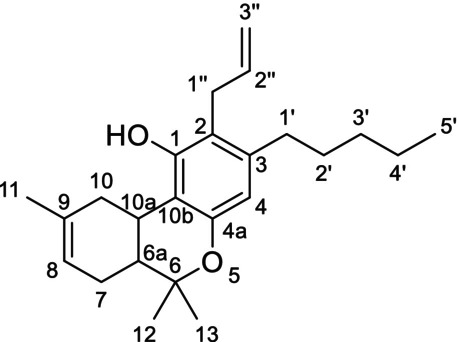	
	[2‐(*E*)‐propen‐1‐yl]‐Δ^8^‐tetrahydrocannabinol‐acetate		[2‐propen‐2‐yl]‐Δ^9^‐tetrahydrocannabinol		[2‐propen‐2‐yl]‐Δ^8^‐tetrahydrocannabinol	
	HRMS (ESI):		HRMS (ESI):		HRMS (ESI):	
	*m/z* calcd for C_26_H_37_O_3_^+^ (M + H)^+^		*m/z* calcd for C_24_H_33_O_2_^+^ (M + H)^+^		*m/z* calcd for C_24_H_33_O_2_^+^ (M + H)^+^	
	397.2737		355.2632		355.2632	
	Found 397.2740		Found 355.2643		Found 355.2640	
	^1^H‐NMR (ppm)	^13^C‐NMR (ppm)	^1^H‐NMR (ppm)	^13^C‐NMR (ppm)	^1^H‐NMR (ppm)	^13^C‐NMR (ppm)
1	—	147.16	—	153.49	—	154.35
2	—	141.81	—	114.11	—	114.13
3	—	123.02	—	140.69	—	140.61
4	6.57 (1H, s)	115.70	6.30 (1H, s)	110.67	6.31 (1H, s)	110.85
4a	—	152.78	—	152.89	—	152.90
6	—	78.62	—	77.01	—	76.61
6a	1.67 (1H, m)	46.01	1.69 (m)	45.80	1.79 (1H, m)	45.30
7	1.90 (1H, m)/1.39 (1H, m)	25.41	1.90 (1H, m)/1.39 (1H, m)	25.10	2.14 (1H, m)/1.81 (1H, m)	36.28
8	6.04 (1H, brd)	123.86	2.17 (2H, m)	31.23	5.42 (1H, m)	119.45
9	—	135.42	—	134.29	—	135.04
10	2.14 (2H, m)	31.45	6.27 (1H, m)	124.06	3.20 (1H, dd, *J* = 16.0, 4.4 Hz)/1.81 (1H, m)	28.11
10a	3.03 (1H, d, *J* = 11.5 Hz)	34.80	3.20 (1H, d, *J* = 10.8 Hz)	33.55	2.70 (1H, td, *J* = 10.9, 4.6 Hz)	32.00
10b	—	115.52	—	114.11	—	114.13
11	1.67 (3H, s)	23.54	1.67 (3H, s)	22.98	1.70 (3H, s)	23.66
12	1.39 (3H, s)	27.93	1.41 (3H, s)	27.58	1.37 (3H, s)	27.70
13	1.08 (3H, s)	19.01	1.09 (3H, s)	19.21	1.10 (3H, s)	18.64
1′	2.44 (2H, td, *J* = 8.3 Hz, 2.1 Hz)	33.68	2.48 (2H, m)	33.35	2.48 (2H, tt, *J* = 14.0, 6.9 Hz)	33.85
2′	1.51 (2H, m)	30.34	1.50 (2H,)	30.73	1.51 (2H, m)	31.03
3′	1.29 (2H, m)	31.83	1.31 (2H, m)	31.51	1.32 (2H, m)	32.04
4′	1.30 (2H, m)	23.10	1.31 (2H, m)	22.57	1.32 (2H, m)	22.71
5′	0.88 (3H, t, *J* = 7.1 Hz)	14.38	0.88 (3H, m)	14.04	0.89 (3H, m)	14.18
1″	6.15 (1H, dq, *J* = 15.9, 1.7 Hz)	124.24	3.37 (2H, dq, *J* = 6.0, 1.6 Hz),	30.52	3.36 (2H, dt, *J* = 5.9, 1.9 Hz)	30.82
2″	5.81 (1H, dq, *J* = 15.9, 6.5 Hz)	129.99	5.98 (1H, m)	136.91	5.99 (1H, m)	136.90
3″	1.85 (3H, dd, *J* = 6.5, 1.8 Hz,)	19.39	5.18 (1H, dq, *J* = 6.8, 1.8 Hz)/5.14 (1H, m)	116.17	5.19 (1H, m)/5.15 (1H, dq, *J* = 8.3, 1.6 Hz)	116.48
Acetyl‐CH_3_	2.23 (3H, s)	21.62	—	—	—	—
C=O	—	170.01	—	—	—	—
‐OH	—	—	5.15 (1H, s)	—	5.10 (1H, s)	—

^a^ Commercial names of the compounds are as provided from online or physical stores.

#### 3.3.2 [2‐Propen‐2‐yl]‐Δ^9^‐Tetrahydrocannabinol

Regarding the major compound of the “tresconol” mixture, we followed the next steps for the structure elucidation. In the aromatic/vinylic region of the ^1^H‐NMR spectra, a singlet at 6.30 ppm (H‐4), a triplet peak at 6.27 ppm (H‐10), a multiplet at 5.99 ppm (H‐2″), a doublet of quartets at 5.18 ppm (H‐3″), and peak at 5.14 ppm (H‐3″) overlapped with the proton of the hydroxyl group were detected. COSY NMR analysis showed a correlation between the protons at 5.18/5.14 ppm and the proton at 5.99 ppm and between the doublet peak at 3.37 ppm (H‐1″) and the multiplet peak at 5.99 ppm. HSQC‐DEPT NMR analysis revealed a double bond carbon with two protons at 116.17 (C‐3″) ppm correlating with the protons at 5.18/5.14 ppm and a secondary carbon at 30.42 (C‐1″) correlating with the methylene protons at 3.37 ppm (H‐1″). From HMBC, NMR analysis correlations between the methylene protons at 3.37 ppm and four different carbons in the aromatic/vinylic region of the spectra at 114.11, 136.91, 140.69, and 153.49 ppm revealed the presence of an allyl group substituted in the aromatic ring of the terpenophenolic cannabinoid skeleton. Additionally, a correlation between protons H‐1″ at 3.37 ppm and C‐1 at 153.49 ppm and a correlation between the methyl proton H‐13 at 1.39 ppm and C‐4a at 152.89 ppm revealed that the allyl group was located at C‐2 (Figure 1). The rest of the spectrum peaks were almost identical with Δ^9^‐THC spectra, especially with (6a*R*,10a*R*)‐Δ^9^‐THC spectra, with the difference that the aromatic singlet peak at 6.30 ppm (H‐4) was more deshielded than the 6.27 ppm (H‐10) double bond peak [15, 16]. Because these compounds are surely made from Δ^8^‐ and Δ^9^‐THC, the absolute configuration should remain unchanged (6a*R*,10a*R*). By 1D and 2D NMR analyses, “tresconol” was identified as the compound [2‐propen‐2‐yl]‐Δ^9^‐tetrahydrocannabinol (IUPAC name: (6a*R*,10a*R*)‐2‐allyl‐6,6,9‐trimethyl‐3‐pentyl‐6a,7,8,10a‐tetrahydro‐6*H*‐benzo[*c*]chromen‐1‐ol) (Table 1) (Figures S20–S24). A *Reaxys* literature search resulted in no result for [2‐propen‐2‐yl]‐Δ^9^‐tetrahydrocannabinol‐acetate, indicating the novelty of this compound, previously unidentified in both literature and the *Cannabis* plant.

#### 3.3.3 [2‐Propen‐2‐yl]‐Δ^8^‐Tetrahydrocannabinol

A similar analysis was carried out for the isolated compound of the commercially available SSC containing product “CBx”. The ^1^H‐NMR spectra of the major compound of “CBx” were similar with [2‐propen‐2‐yl]‐Δ^9^‐tetrahydrocannabinol spectra with the double bond peak from 6.27 (H‐10) ppm, moving to 5.42 ppm (H‐8), resembling the major difference of Δ^9^‐THC and Δ^8^‐THC ^1^H‐NMR spectra [14]. The allyl group was located at C‐2 based on the HMBC NMR correlation between protons H‐1″ at 3.36 ppm and C‐1 at 154.35 ppm and a weak *J* ^4^ correlation between the methyl proton H‐12 at 1.37 ppm and C‐4a at 152.90 ppm (Figures 1 and S25–S29). One‐ and two‐dimensional analyses ensured that the major compound of “CBx” mixture was [2‐propen‐2‐yl]‐Δ^8^‐tetrahydrocannabinol (IUPAC name: (6a*R*,10a*R*)‐2‐allyl‐6,6,9‐trimethyl‐3‐pentyl‐6a,7,10,10a‐tetrahydro‐6*H*‐benzo[*c*]chromen‐1‐ol) (Table 1) (Figures S31–S34). [2‐propen‐2‐yl]‐Δ^9^‐tetrahydrocannabinol was also identified in the “CBx” mixture (Figure S29). The synthesis of [2‐propen‐2‐yl]‐Δ^8^‐tetrahydrocannabinol is described in a patent application (EP0279308) [17] and relies on the well‐known Claisen rearrangement of allylphenol ethers to *ortho*‐allylphenols and thus explains the ready availability of such 2‐alkenyl‐substituted derivatives of THC in one simple step (Figure S36). However, there are no spectroscopic or chromatographic data available for this compound making the identification of its presence in commercial products very difficult.

In general, these compounds can be synthesized using affordable, readily available chemicals in most organic synthesis laboratories. These compounds bear a close resemblance to the psychoactive cannabinoids Δ^9^‐THC and Δ^8^‐THC, suggesting they are likely to maintain cannabinoid receptor 1 affinity and exhibit psychotropic properties upon administration, similar to other THC‐like cannabinoids [18]. Additionally, Edery et al. [19] demonstrated that [2‐ethyl]‐Δ^8^‐THC, a structurally similar analog to the identified compounds, exhibited significant cannabimimetic effects in rhesus monkeys, suggesting that the previously unknown identified SSCs may also possess similar pharmacological properties and warrant further investigation into their potential behavioral impacts. Moreover, their enhanced lipophilicity (logP = 6.5) compared to their precursors Δ^9^‐THC and Δ^8^‐THC (logP = 5.5) may lead to increased blood–brain barrier penetration. Notably, these compounds have never been identified in *Cannabis* plants, and the lack of available standards makes the identification and quantitation by the standard forensic analytical methods very difficult. These products consist of complex mixtures of previously unknown or inadequately described synthetic cannabinoids without established toxicological or pharmacological safety profiles, and based on the existing literature, there are no other documented natural cannabinoids with allyl and/or propenyl groups [20]. Additionally, they might contain various synthetic byproducts and the residues from the chemicals or solvents used in their synthesis, because they are not produced under regulated conditions.

## 4 Conclusion

Three SSCs were identified in commercially available products with limited pharmacological and toxicological data: [2‐(*E*)‐propen‐1‐yl]‐Δ^8^‐tetrahydrocannabinol‐acetate in “CB9”, [2‐propen‐2‐yl]‐Δ^9^‐tetrahydrocannabinol in “tresconol” and [2‐propen‐2‐yl]‐Δ^8^‐tetrahydrocannabinol in “CBx”. These unregulated compounds were shown to be detectable using GC‐MS and NMR, demonstrating the usefulness of these techniques for forensic identification. The presence of these SSCs, which bear structural similarities to known psychoactive cannabinoids like Δ^8^‐ and Δ^9^‐THC, raises significant public health concerns due to the potential for psychoactive effects and the lack of established safety profiles.

These findings, in parallel with similar studies, highlight the need for regulatory authorities to prioritize the monitoring and control of these SSC to mitigate potential health risks, as the market for such compounds continues to expand [21]. The compounds' prevalence in consumer products, often marketed as natural and hemp‐derived, highlights the innovative approaches used to bypass current legislation. Further toxicological and pharmacological research is essential to fully understand the impact of these substances on public health, particularly among younger users. Regulatory frameworks must evolve to include these emerging SSC.

## Disclosure

V. M. is the owner of Ekati Alchemy Lab (Spain), no competing financial interests exist.

## Conflicts of Interest

The authors declare no conflicts of interest.

## Supporting information


**Figure S1** GC‐MS chromatogram of the "CB9" mixture and the EI mass spectra of the main detected peaks (> 5%).
**Figure S2** GC‐MS chromatogram of the "tresconol" mixture and the EI mass spectra of the main detected peaks (> 5%).
**Figure S3** GC‐MS chromatogram of the "CBx" mixture and the EI mass spectra of the main detected peaks (> 5%).
**Figure S4** GC‐MS chromatogram of "CB9" mixture (1).
**Figure S5** GC‐MS chromatogram of "CB9" mixture (2).
**Figure S6** EI mass spectra of [2‐(*E*)‐propen‐1‐yl]‐Δ^8^‐tetrahydrocannabinol‐acetate, [M]^.+^= 396.3.
**Figure S7** ^1^H NMR spectra of “CB9” mixture, in CDCl_3_, 400 MHz.
**Figure S8** Preparative HPLC trace for the isolation of the major peak from the “CB9” mixture.
**Figure S9** ^1^H NMR spectra of "[2‐(*E*)‐propen‐1‐yl]‐Δ^8^‐tetrahydrocannabinol‐acetate", in CDCl_3_, 400 MHz.
**Figure S10** ^13^C NMR spectra of "[2‐(*E*)‐propen‐1‐yl]‐Δ^8^‐tetrahydrocannabinol‐acetate", in CDCl_3_, 100 MHz.
**Figure S11** ^1^H‐^13^C HSQC‐DEPT NMR spectra of "[2‐(*E*)‐propen‐1‐yl]‐Δ^8^‐tetrahydrocannabinol‐acetate", in CDCl_3_.
**Figure S12** ^1^H‐^13^C HMBC NMR spectra of "[2‐(*E*)‐propen‐1‐yl]‐Δ^8^‐tetrahydrocannabinol‐acetate", in CDCl_3_.
**Figure S13** ^1^H‐ ^1^H COSY spectra of "[2‐(*E*)‐propen‐1‐yl]‐Δ8‐tetrahydrocannabinol‐acetate", in CDCl_3._
**Figure S14** Structure of "[2‐(*E*)‐propen‐1‐yl]‐Δ^8^‐tetrahydrocannabinol‐acetate".
**Figure S15** GC‐MS chromatogram of "tresconol" mixture (1).
**Figure S16** GC‐MS chromatogram of "tresconol" mixture (2).
**Figure S17** EI mass spectra of “[2‐propen‐2‐yl]‐Δ^9^‐tetrahydrocannabinol”, [M]^.+^= 354.2.
**Figure S18** ^1^H NMR spectra of "tresconol", in CDCl_3_, 400 MHz.
**Figure S19** Preparative HPLC trace for the isolation of the major peak from the "tresconol" mixture.
**Figure S20** ^1^H NMR spectra of "[2‐propen‐2‐yl]‐Δ^9^‐tetrahydrocannabinol", in CDCl_3_, 400 MHz.
**Figure S21** ^13^C NMR spectra of "[2‐propen‐2‐yl]‐Δ^9^‐tetrahydrocannabinol", in CDCl_3_, 100 MHz.
**Figure S22** ^1^H‐ ^13^C HMQC‐DEPT NMR spectra of "[2‐propen‐2‐yl]‐Δ^9^‐tetrahydrocannabinol", in CDCl_3_.
**Figure S23** ^1^H‐ ^13^C HMBC NMR spectra of "[2‐propen‐2‐yl]‐Δ^9^‐tetrahydrocannabinol", in CDCl_3_.
**Figure S24** ^1^H‐ ^1^H COSY NMR spectra of "[2‐propen‐2‐yl]‐Δ^9^‐tetrahydrocannabinol", in CDCl_3_.
**Figure S25** Structure of "[2‐propen‐2‐yl]‐Δ^9^‐tetrahydrocannabinol".
**Figure S26** GC‐MS chromatogram of "CBx" mixture (1).
**Figure S27** GC‐MS chromatogram of "CBx" mixture (2).
**Figure S28** EI mass spectra of “[2‐propen‐2‐yl]‐Δ^8^‐tetrahydrocannabinol”, [M]^+^= 354.2.
**Figure S29** ^1^H NMR spectra of "CBx", in CDCl_3_, 400 MHz.
**Figure S30** Preparative HPLC trace for the isolation of the major peak from the “CBx” mixture.
**Figure S31** ^1^H NMR spectra of "[2‐propen‐2‐yl]‐Δ^8^‐tetrahydrocannabinol", in CDCl_3_, 400 MHz.
**Figure S32** ^13^C NMR spectra of "[2‐propen‐2‐yl]‐Δ^8^‐tetrahydrocannabinol", in CDCl_3_, 100 MHz.
**Figure S33** ^1^H‐ ^13^C HMQC‐DEPT NMR spectra of "[2‐propen‐2‐yl]‐Δ^8^‐tetrahydrocannabinol", in CDCl_3_.
**Figure S34** ^1^H‐ ^13^C HMBC NMR spectra of "[2‐propen‐2‐yl]‐Δ^8^‐tetrahydrocannabinol", in CDCl_3_.
**Figure S35** Structure of "[2‐propen‐2‐yl]‐Δ^8^‐tetrahydrocannabinol".
**Figure S36** Schematic representation of "[2‐propen‐2‐yl]‐Δ^8^‐tetrahydrocannabinol" synthesis based on the patent EP0279308A2.

## Data Availability

The data that support the findings of this study are available in the Supporting Information of this article.
