# Supplementary material for: Identification of Three Novel Tetrahydrocannabinol Analogs in the European Market
Source: Drug Test Anal. 2025 Feb 6;17(9):1594–600. doi: 10.1002/dta.3866 (PMC12401631; doi:10.1002/dta.3866)
Supplement: Supplementary file 1 — Figure S1 GC‐MS chromatogram of the "CB9" mixture and the EI mass spectra of the main detected peaks (> 5%). Figure S2 GC‐MS chromatogram of the "tresconol" mixture and the EI mass spectra of the main detected peaks (> 5%). Figure S3 GC‐MS chromatogram of the "CBx" mixture and the EI mass spectra of the main detected peaks (> 5%). Figure S4 GC‐MS chromatogram of "CB9" mixture (1). Figure S5 GC‐MS chromatogram of "CB9" mixture (2). Figure S6 EI mass spectra of [2‐(E)‐propen‐1‐yl]‐Δ8‐tetrahydrocannabinol‐acetate, [M].+= 396.3. Figure S7 1H NMR spectra of “CB9” mixture, in CDCl3, 400 MHz. Figure S8 Preparative HPLC trace for the isolation of the major peak from the “CB9” mixture. Figure S9 1H NMR spectra of "[2‐(E)‐propen‐1‐yl]‐Δ8‐tetrahydrocannabinol‐acetate", in CDCl3, 400 MHz. Figure S10 13C NMR spectra of "[2‐(E)‐propen‐1‐yl]‐Δ8‐tetrahydrocannabinol‐acetate", in CDCl3, 100 MHz. Figure S11 1H‐13C HSQC‐DEPT NMR spectra of "[2‐(E)‐propen‐1‐yl]‐Δ8‐tetrahydrocannabinol‐acetate", in CDCl3. Figure S12 1H‐13C HMBC NMR spectra of "[2‐(E)‐propen‐1‐yl]‐Δ8‐tetrahydrocannabinol‐acetate", in CDCl3. Figure S13 1H‐ 1H COSY spectra of "[2‐(E)‐propen‐1‐yl]‐Δ8‐tetrahydrocannabinol‐acetate", in CDCl3. Figure S14 Structure of "[2‐(E)‐propen‐1‐yl]‐Δ8‐tetrahydrocannabinol‐acetate". Figure S15 GC‐MS chromatogram of "tresconol" mixture (1). Figure S16 GC‐MS chromatogram of "tresconol" mixture (2). Figure S17 EI mass spectra of “[2‐propen‐2‐yl]‐Δ9‐tetrahydrocannabinol”, [M].+= 354.2. Figure S18 1H NMR spectra of "tresconol", in CDCl3, 400 MHz. Figure S19 Preparative HPLC trace for the isolation of the major peak from the "tresconol" mixture. Figure S20 1H NMR spectra of "[2‐propen‐2‐yl]‐Δ9‐tetrahydrocannabinol", in CDCl3, 400 MHz. Figure S21 13C NMR spectra of "[2‐propen‐2‐yl]‐Δ9‐tetrahydrocannabinol", in CDCl3, 100 MHz. Figure S22 1H‐ 13C HMQC‐DEPT NMR spectra of "[2‐propen‐2‐yl]‐Δ9‐tetrahydrocannabinol", in CDCl3. Figure S23 1H‐ 13C HMBC NMR spectra of "[2‐propen‐2‐yl]‐Δ9‐tetrahydrocannab [file DTA-17-1594-s001.docx]

**Supplementary material**

**Identification of Three Novel Tetrahydrocannabinol analogues in the European Market**

Evangelos Dadiotis^a^, Sotiris Mpakaoukas^a^, Vangelis Mitsis^b^, Eleni Melliou^a^, Prokopios Magiatis^a,*^

^a^Laboratory of Pharmacognosy and Natural Products Chemistry, Department of Pharmacy, National and Kapodistrian University of Athens, Panepistimioupolis Zografou, 15771 Athens, Greece

^b^Ekati Alchemy Lab SL, 08180 Moià, Barcelona, Spain

**Table of Figures**

[Figure S1: GC-MS chromatogram of the "CB9" mixture with EI mass spectra for the main detected peaks 6](#_Toc182257151)

[Figure S2: GC-MS chromatogram of the "tresconol" mixture with EI mass spectra for the main detected peaks. 9](#_Toc182257152)

[Figure S3: GC-MS chromatogram of the "CBx" mixture with EI mass spectra for the main detected peaks. 15](#_Toc182257153)

[Figure S4: GC-MS chromatogram of "CB9" mixture (1). 16](#_Toc182257154)

[Figure S5: GC-MS chromatogram of "CB9" mixture (2). 16](#_Toc182257155)

[Figure S6: EI mass spectra of [2-(*E*)-propen-1-yl]-Δ^8^-tetrahydrocannabinol-acetate, [M]^.+^= 396.3. 17](#_Toc182257156)

[Figure S7: ^1^H NMR spectra of “CB9” mixture, in CDCl_3_, 400 MHz. 17](#_Toc182257157)

[Figure S8: Preparative HPLC trace for the isolation of the major peak from the “CB9” mixture 18](#_Toc182257158)

[Figure S9: ^1^H NMR spectra of "[2-(*E*)-propen-1-yl]-Δ^8^-tetrahydrocannabinol-acetate", in CDCl_3_, 400 MHz. 19](#_Toc182257159)

[Figure S10: ^13^C NMR spectra of "[2-(*E*)-propen-1-yl]-Δ^8^-tetrahydrocannabinol-acetate", in CDCl_3_, 100 MHz. 20](#_Toc182257160)

[Figure S11:^1^H-^13^C HSQC-DEPT NMR spectra of "[2-(*E*)-propen-1-yl]-Δ^8^-tetrahydrocannabinol-acetate", in CDCl_3_. 21](#_Toc182257161)

[Figure S12: ^1^H-^13^C HMBC NMR spectra of "[2-(*E*)-propen-1-yl]-Δ^8^-tetrahydrocannabinol-acetate", in CDCl_3_. 22](#_Toc182257162)

[Figure S13: ^1^H- ^1^H COSY spectra of "[2-(*E*)-propen-1-yl]-Δ8-tetrahydrocannabinol-acetate", in CDCl_3._ 23](#_Toc182257163)

[Figure S14: Structure of "[2-(*E*)-propen-1-yl]-Δ^8^-tetrahydrocannabinol-acetate". 24](#_Toc182257164)

[Figure S15: GC-MS chromatogram of "tresconol" mixture (1). 25](#_Toc182257165)

[Figure S16: GC-MS chromatogram of "tresconol" mixture (2). 25](#_Toc182257166)

[Figure S17: EI mass spectra of “[2-propen-2-yl]-Δ^9^-tetrahydrocannabinol”, [M]^.+^= 354.2. 26](#_Toc182257167)

[Figure S18: ^1^H NMR spectra of "tresconol", in CDCl_3_, 400 MHz. 26](#_Toc182257168)

[Figure S19: Preparative HPLC trace for the isolation of the major peak from the "tresconol" mixture. 27](#_Toc182257169)

[Figure S20: ^1^H NMR spectra of "[2-propen-2-yl]-Δ9-tetrahydrocannabinol", in CDCl_3_, 400 MHz. 28](#_Toc182257170)

[Figure S21: ^13^C NMR spectra of "[2-propen-2-yl]-Δ^9^-tetrahydrocannabinol", in CDCl_3_, 100 MHz. 29](#_Toc182257171)

[Figure S22: ^1^H- ^13^C HMQC-DEPT NMR spectra of "[2-propen-2-yl]-Δ^9^-tetrahydrocannabinol", in CDCl_3_. 30](#_Toc182257172)

[Figure S23: ^1^H- ^13^C HMBC NMR spectra of "[2-propen-2-yl]-Δ^9^-tetrahydrocannabinol", in CDCl_3_. 31](#_Toc182257173)

[Figure S24: ^1^H- ^1^H COSY NMR spectra of "[2-propen-2-yl]-Δ^9^-tetrahydrocannabinol", in CDCl_3_. 32](#_Toc182257174)

[Figure S25: Structure of "[2-propen-2-yl]-Δ^9^-tetrahydrocannabinol". 33](#_Toc182257175)

[Figure S26: GC-MS chromatogram of "CBx" mixture (1). 34](#_Toc182257176)

[Figure S27: GC-MS chromatogram of "CBx" mixture (2). 34](#_Toc182257177)

[Figure S28: EI mass spectra of “[2-propen-2-yl]-Δ^8^-tetrahydrocannabinol”, [M]^.+^= 354.2. 35](#_Toc182257178)

[Figure S29: ^1^H NMR spectra of "CBx", in CDCl_3_, 400 MHz. 35](#_Toc182257179)

[Figure 30: Preparative HPLC trace for the isolation of the major peak from the “CBx” mixture. 36](#_Toc182257180)

[Figure S31: ^1^H NMR spectra of "[2-propen-2-yl]-Δ^8^-tetrahydrocannabinol", in CDCl_3_, 400 MHz. 37](#_Toc182257181)

[Figure S32: ^13^C NMR spectra of "[2-propen-2-yl]-Δ^8^-tetrahydrocannabinol", in CDCl_3_, 100 MHz. 38](#_Toc182257182)

[Figure S33: ^1^H- ^13^C HMQC-DEPT NMR spectra of "[2-propen-2-yl]-Δ^8^-tetrahydrocannabinol", in CDCl_3_. 39](#_Toc182257183)

[Figure S34: ^1^H- ^13^C HMBC NMR spectra of "[2-propen-2-yl]-Δ^8^-tetrahydrocannabinol", in CDCl_3_. 40](#_Toc182257184)

[Figure S35: Structure of "[2-propen-2-yl]-Δ^8^-tetrahydrocannabinol". 41](#_Toc182257185)

[Figure S36: Schematic representation of "[2-propen-2-yl]-Δ^8^-tetrahydrocannabinol" synthesis based on patent EP0279308A2. 41](#_Toc182257186)

**“CB9” mixture GC-MS analysis** **

Figure S1: GC-MS chromatogram of the "CB9" mixture and the EI mass spectra of the main detected peaks (> 5%)

**“Tresconol” mixture GC-MS analysis**

Figure S2: GC-MS chromatogram of the "tresconol" mixture and the EI mass spectra of the main detected peaks (> 5%).

**“CBx” mixture GC-MS analysis**

************

Figure S3: GC-MS chromatogram of the "CBx" mixture and the EI mass spectra of the main detected peaks (> 5%).

**“CB9” GC-MS and NMR analysis**

Figure S4: GC-MS chromatogram of "CB9" mixture (1).

Figure S5: GC-MS chromatogram of "CB9" mixture (2).

Figure S6: EI mass spectra of [2-(E)-propen-1-yl]-Δ^8^-tetrahydrocannabinol-acetate, [M]^.+^= 396.3.


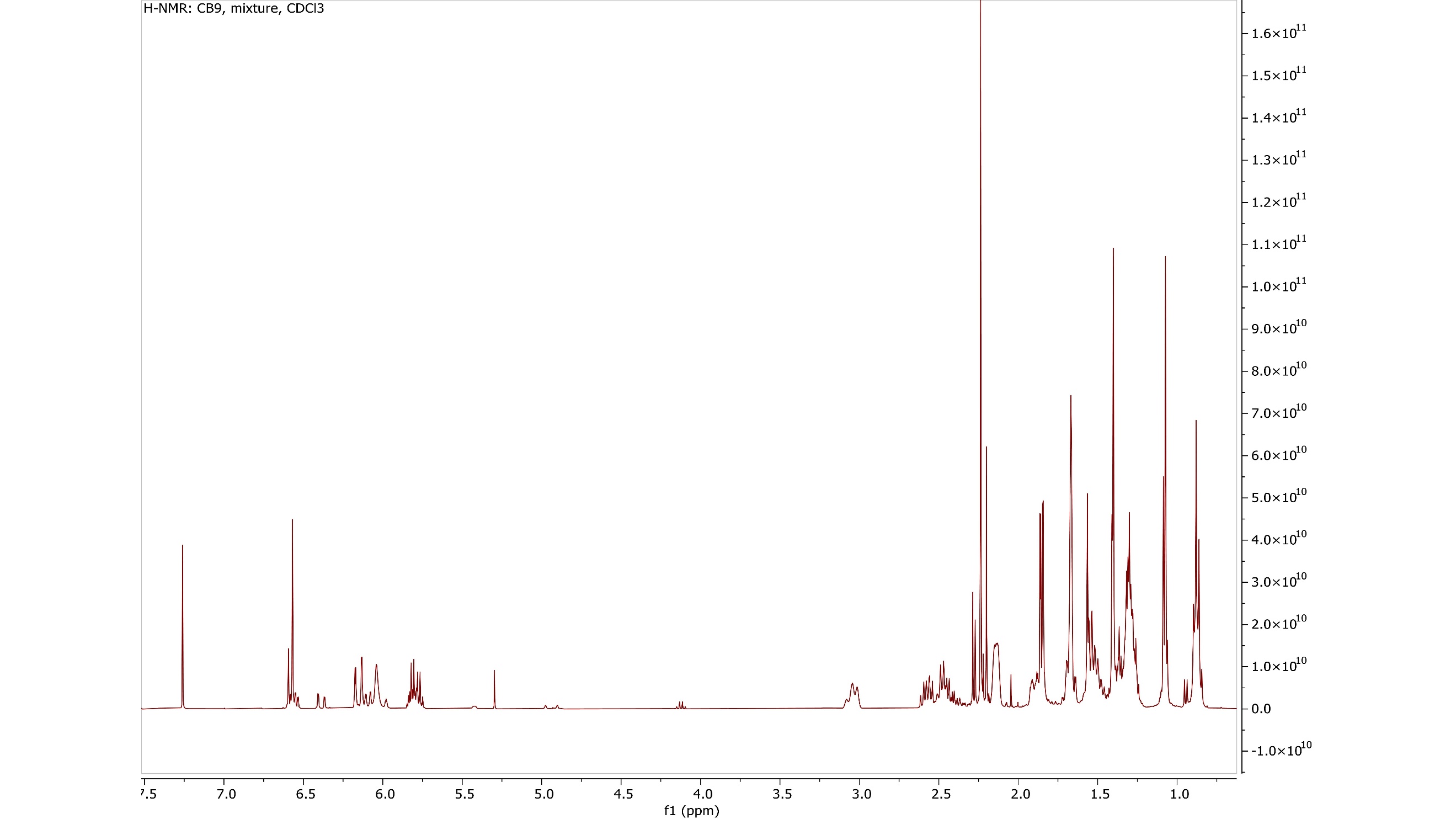


Figure S7: ^1^H NMR spectra of “CB9” mixture, in CDCl_3_, 400 MHz.


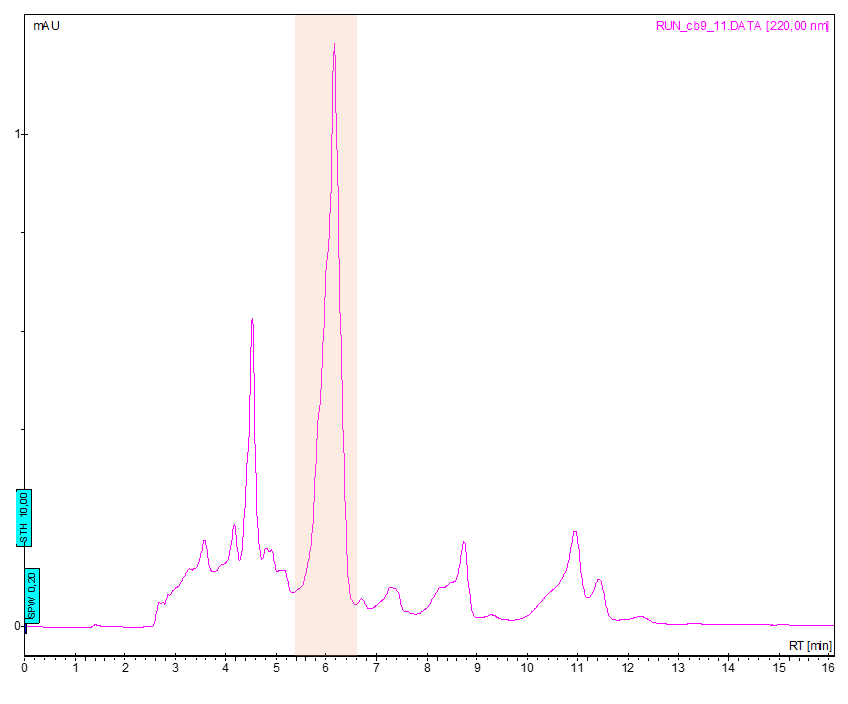


Figure S8: Preparative HPLC trace for the isolation of the major peak from the “CB9” mixture


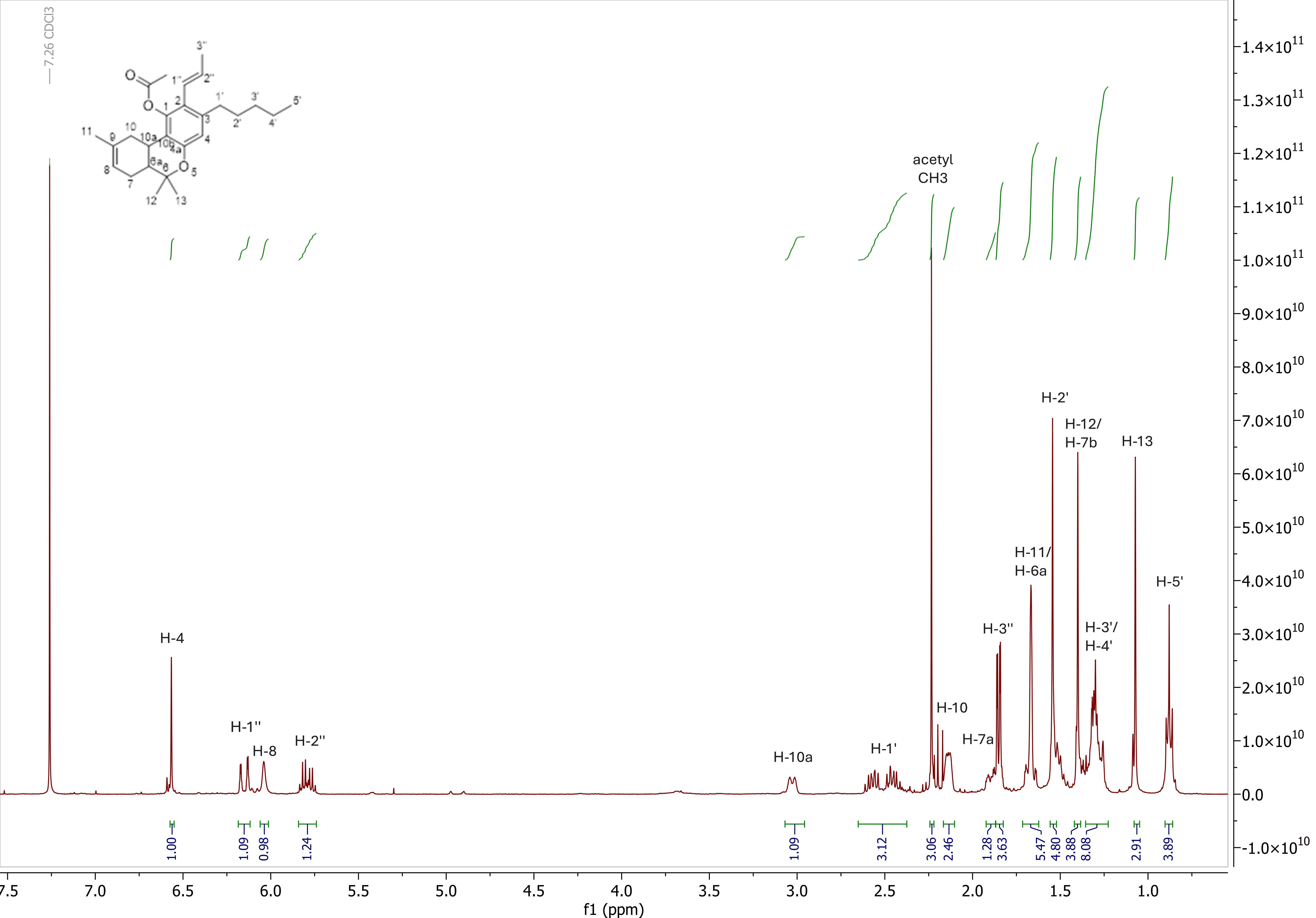


Figure S9: ^1^H NMR spectra of "[2-(E)-propen-1-yl]-Δ^8^-tetrahydrocannabinol-acetate", in CDCl_3_, 400 MHz.


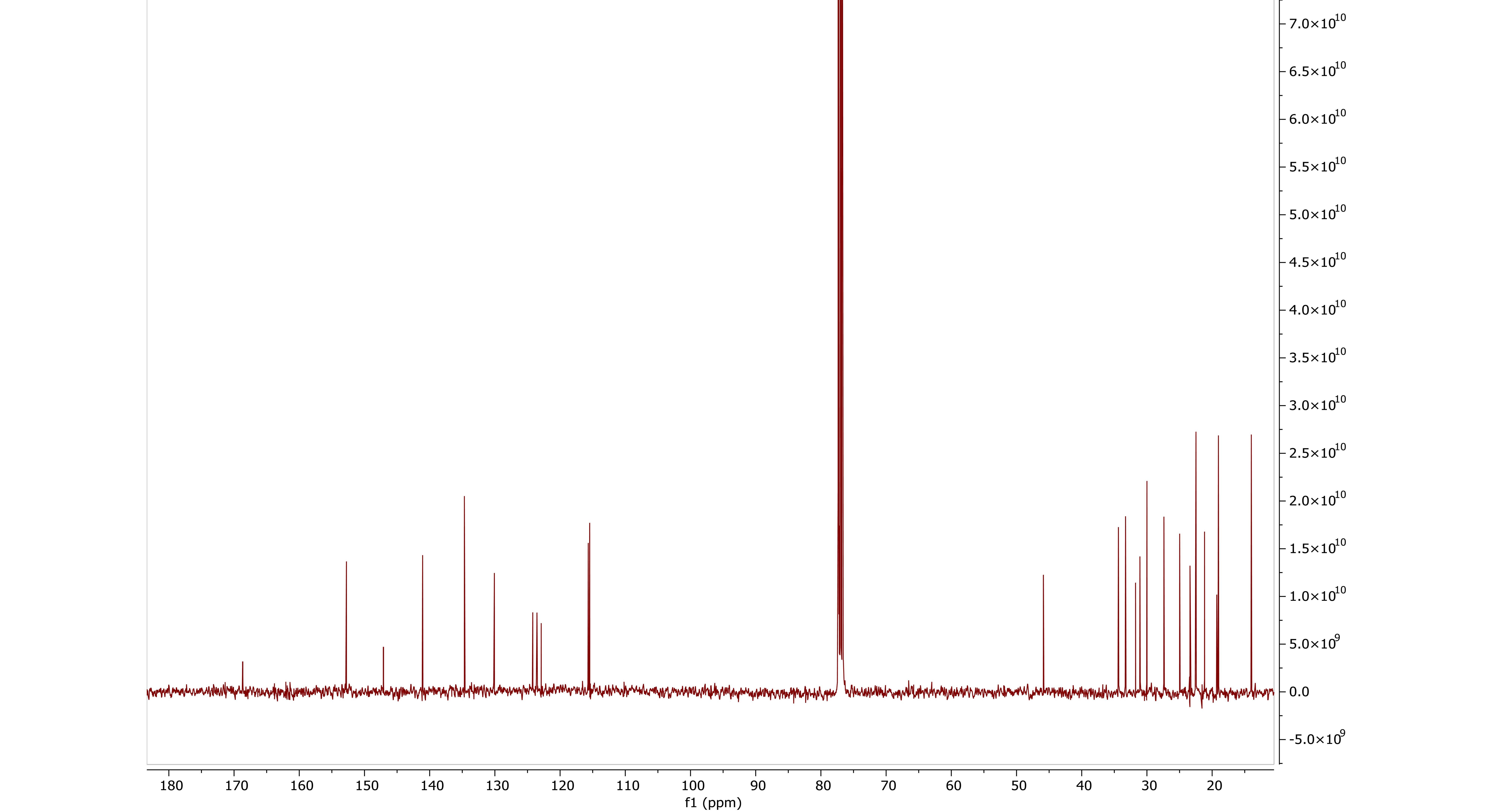


Figure S10: ^13^C NMR spectra of "[2-(E)-propen-1-yl]-Δ^8^-tetrahydrocannabinol-acetate", in CDCl_3_, 100 MHz.


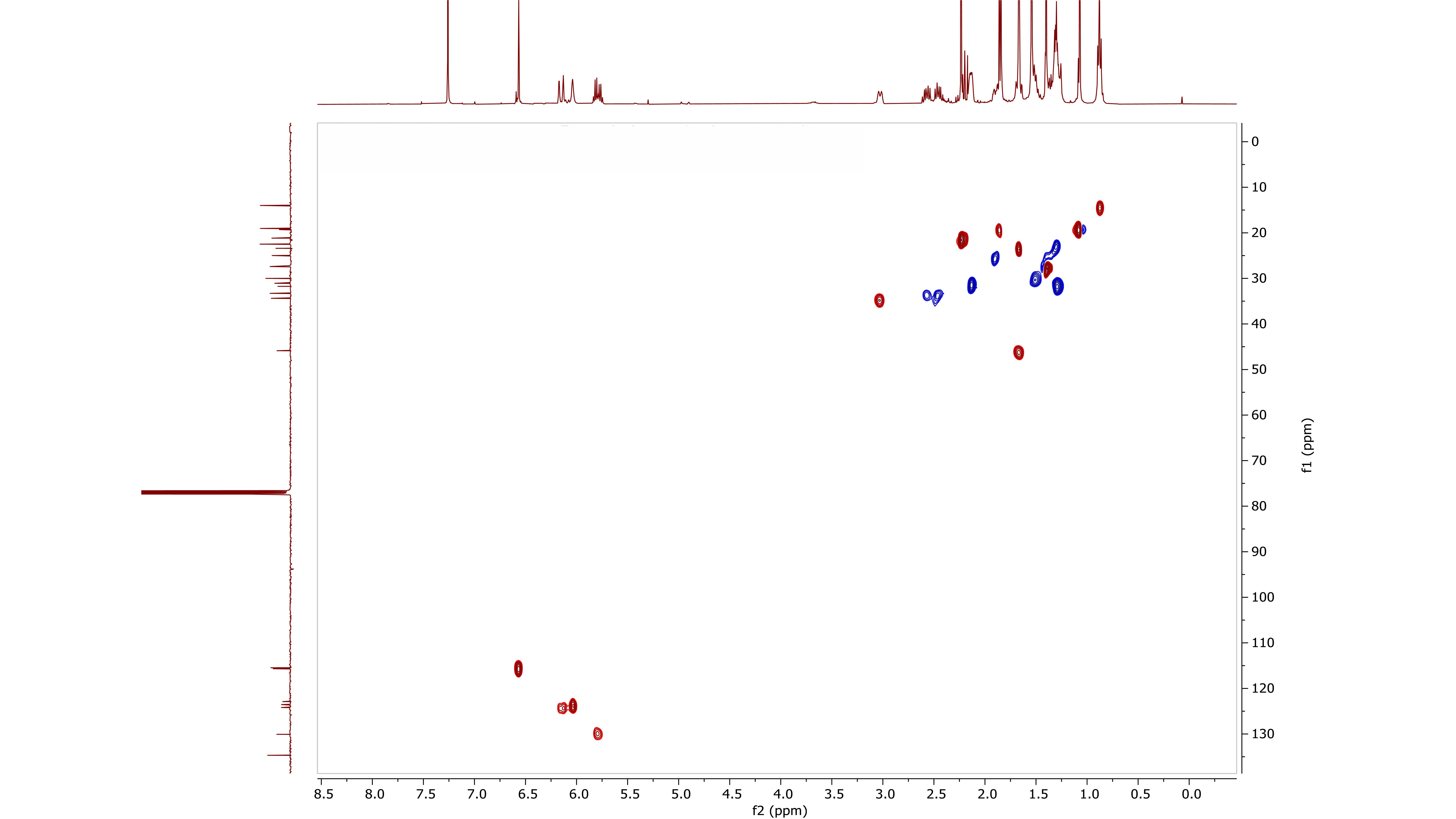


Figure S11:^1^H-^13^C HSQC-DEPT NMR spectra of "[2-(E)-propen-1-yl]-Δ^8^-tetrahydrocannabinol-acetate", in CDCl_3_.

.


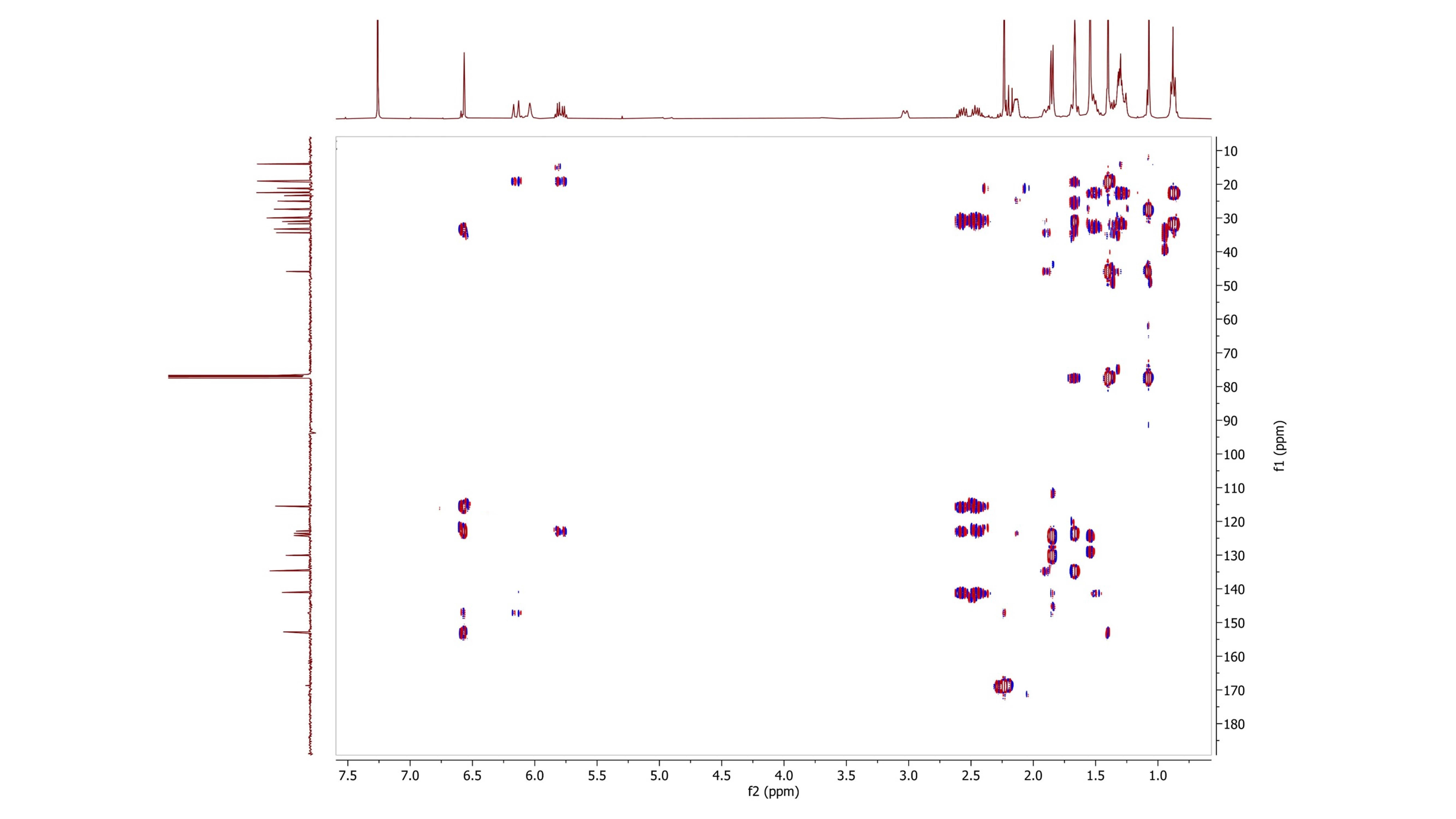


Figure S12: ^1^H-^13^C HMBC NMR spectra of "[2-(E)-propen-1-yl]-Δ^8^-tetrahydrocannabinol-acetate", in CDCl_3_.


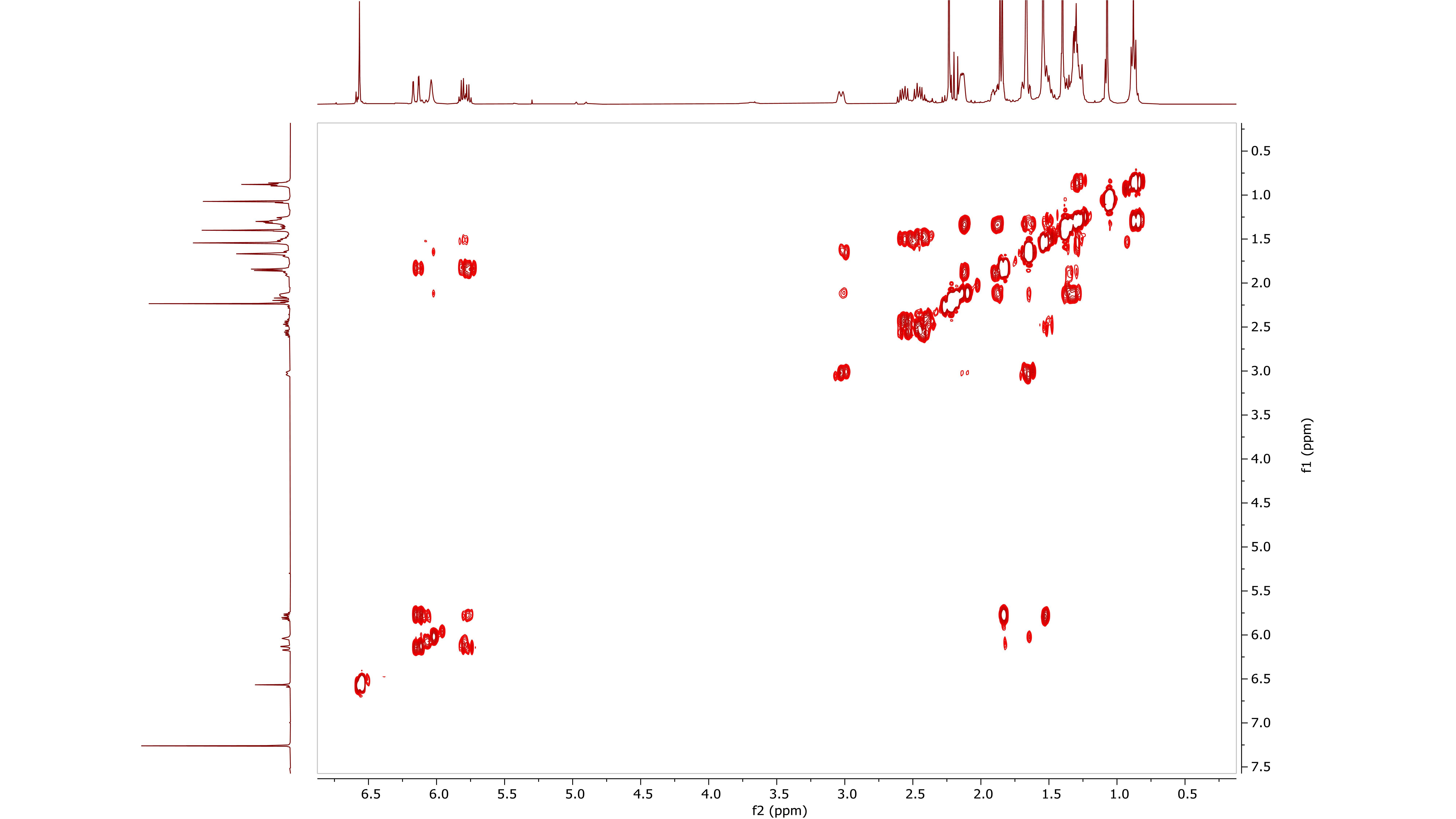


Figure S13: ^1^H- ^1^H COSY spectra of "[2-(E)-propen-1-yl]-Δ8-tetrahydrocannabinol-acetate", in CDCl_3._

Figure S14: Structure of "[2-(E)-propen-1-yl]-Δ^8^-tetrahydrocannabinol-acetate".

**“Tresconol” GC-MS and NMR analysis**

Figure S15: GC-MS chromatogram of "tresconol" mixture (1).

Figure S16: GC-MS chromatogram of "tresconol" mixture (2).

Figure S17: EI mass spectra of “[2-propen-2-yl]-Δ^9^-tetrahydrocannabinol”, [M]^.+^= 354.2.


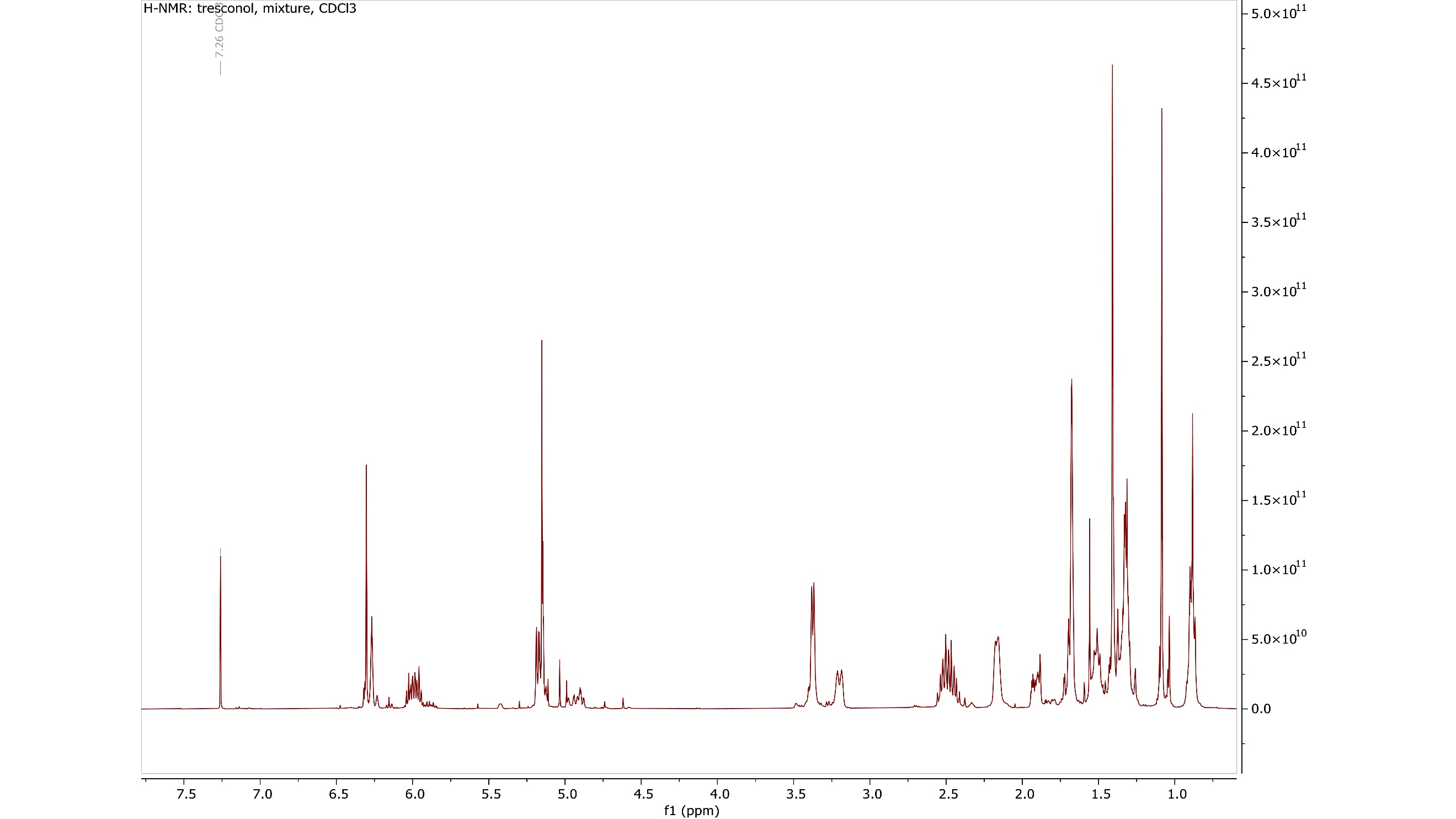


Figure S18: ^1^H NMR spectra of "tresconol", in CDCl_3_, 400 MHz.


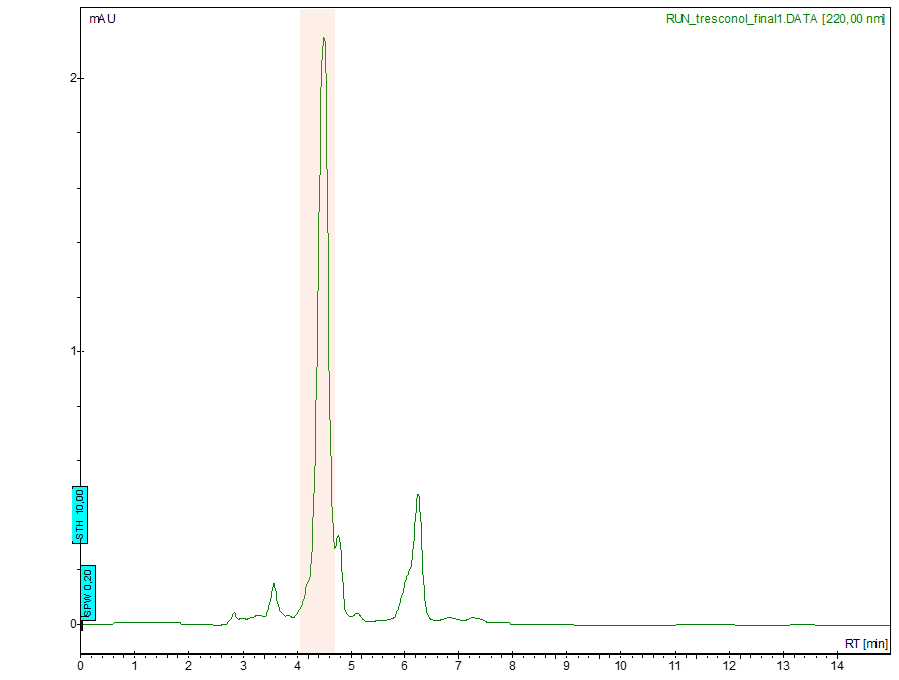


Figure S19: Preparative HPLC trace for the isolation of the major peak from the "tresconol" mixture.


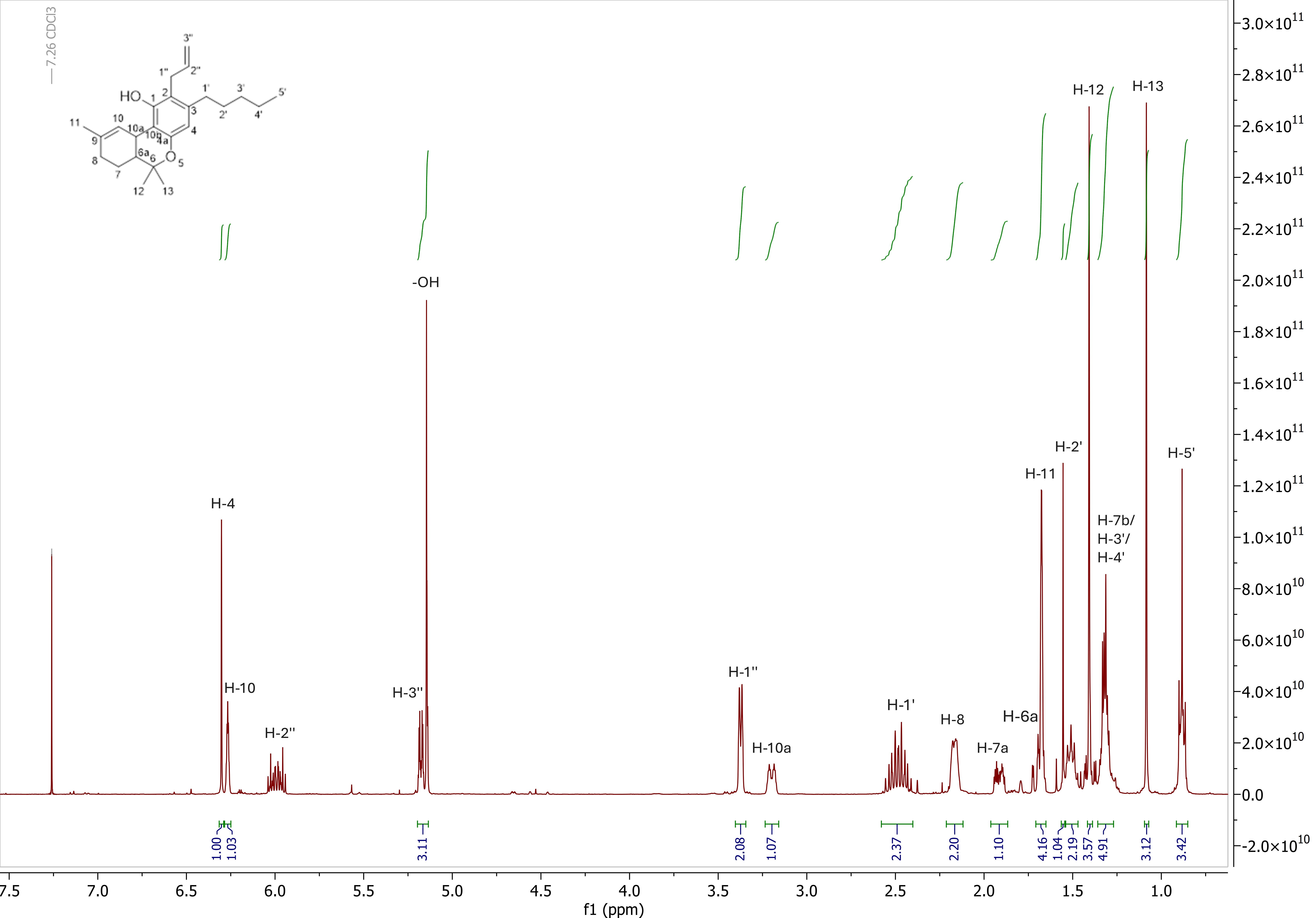


Figure S20: ^1^H NMR spectra of "[2-propen-2-yl]-Δ^9^-tetrahydrocannabinol", in CDCl_3_, 400 MHz.


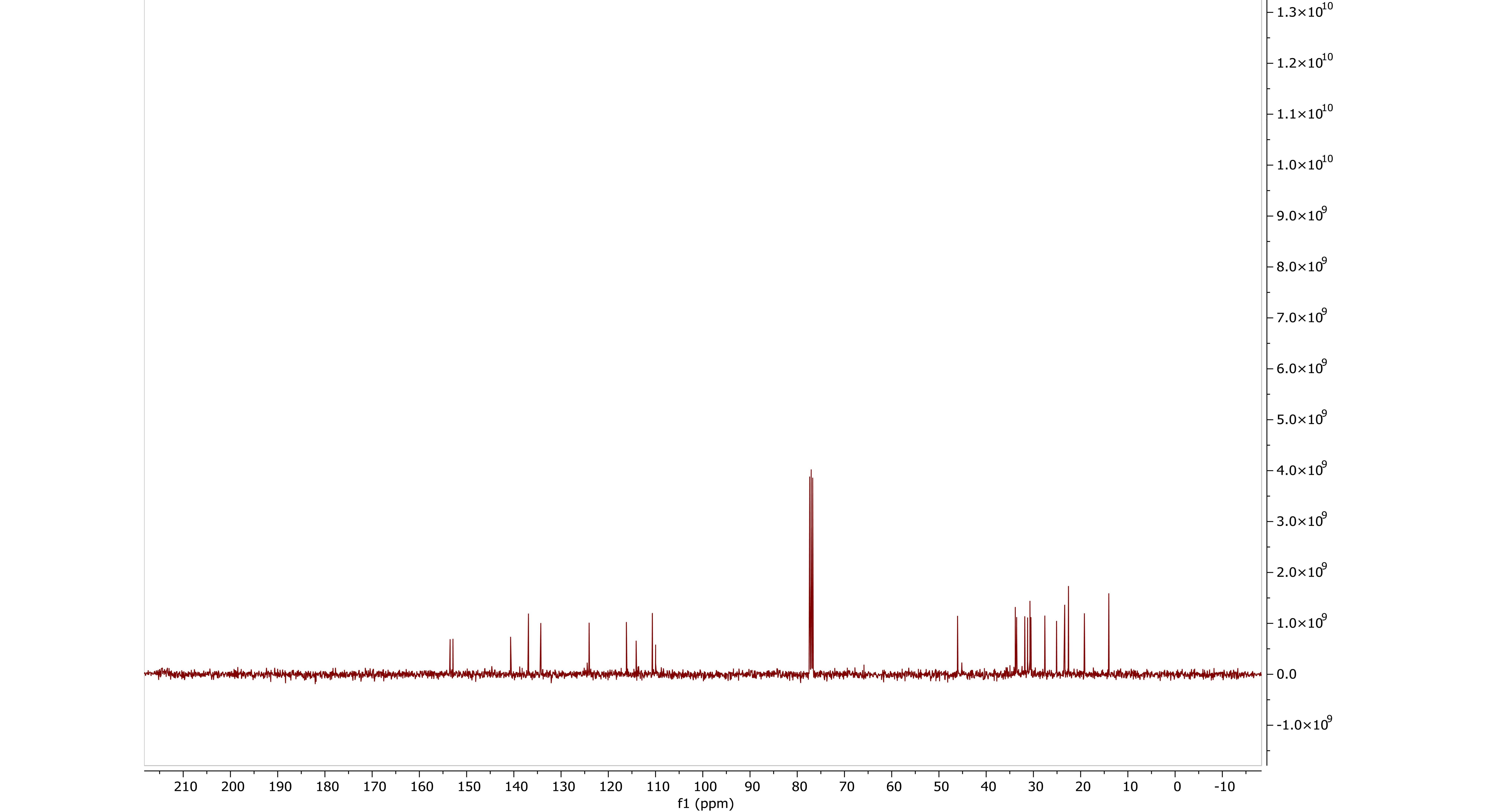


Figure S21: ^13^C NMR spectra of "[2-propen-2-yl]-Δ^9^-tetrahydrocannabinol", in CDCl_3_, 100 MHz.


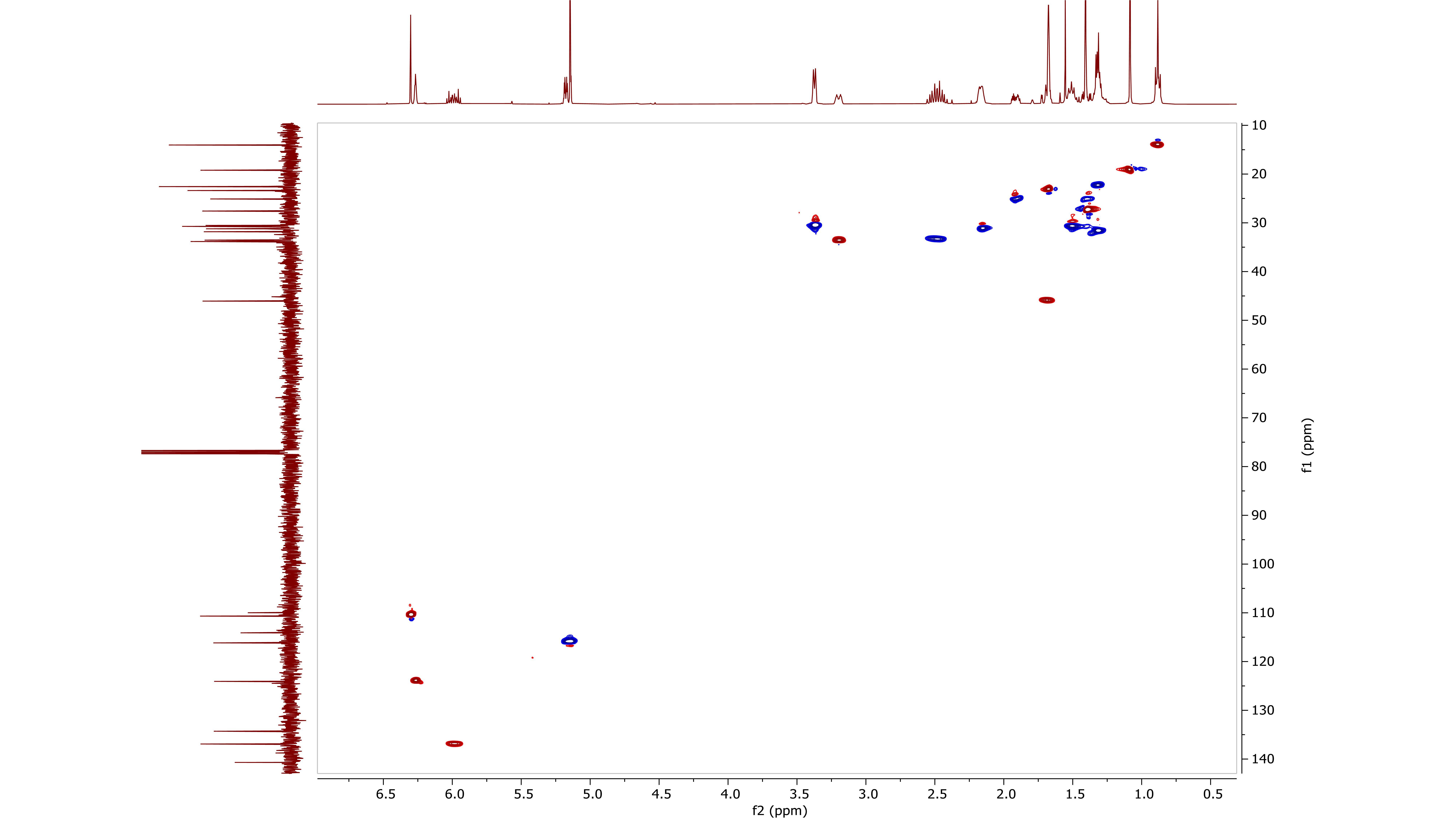


Figure S22: ^1^H- ^13^C HMQC-DEPT NMR spectra of "[2-propen-2-yl]-Δ^9^-tetrahydrocannabinol", in CDCl_3_.


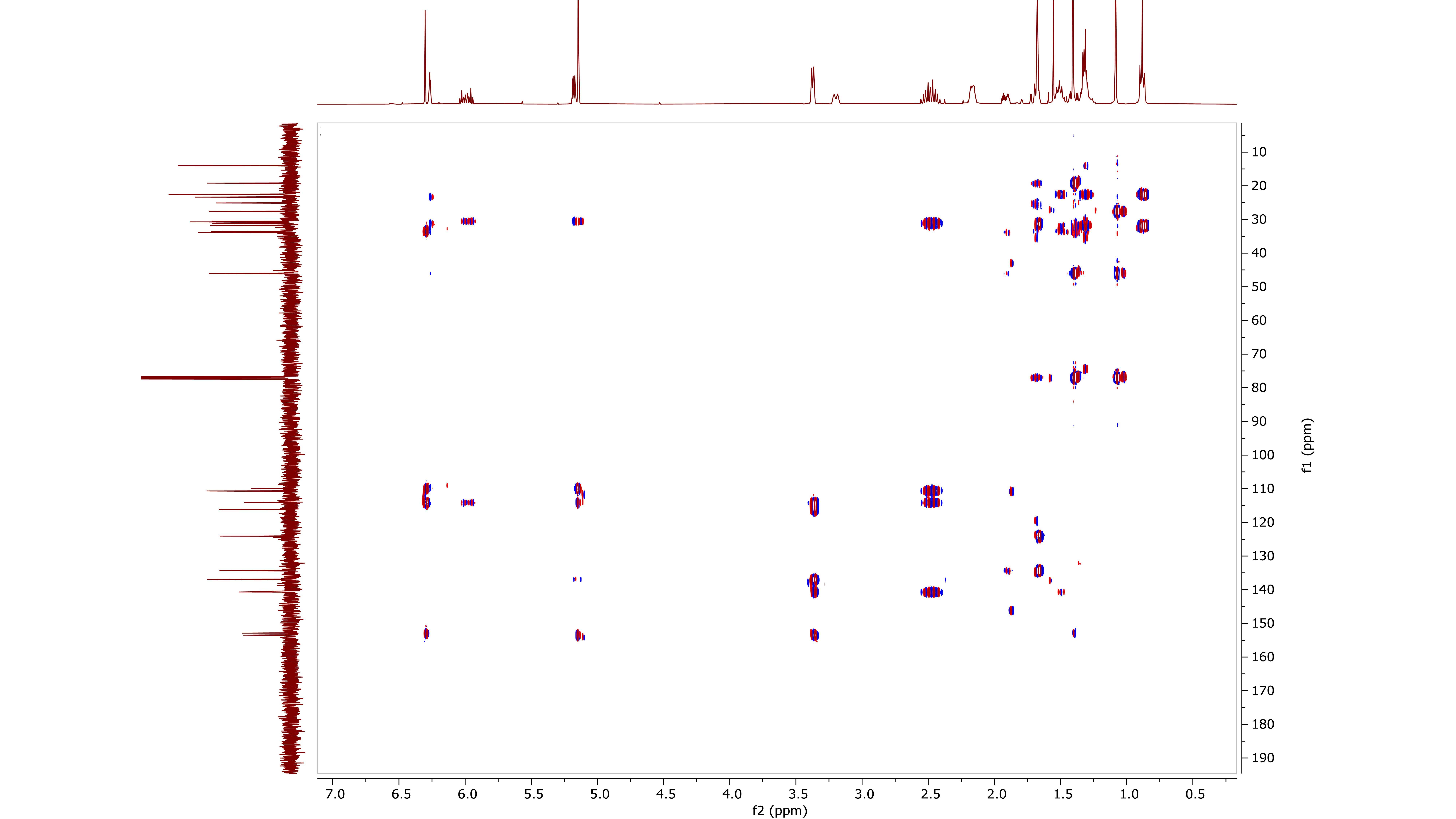


Figure S23: ^1^H- ^13^C HMBC NMR spectra of "[2-propen-2-yl]-Δ^9^-tetrahydrocannabinol", in CDCl_3_.


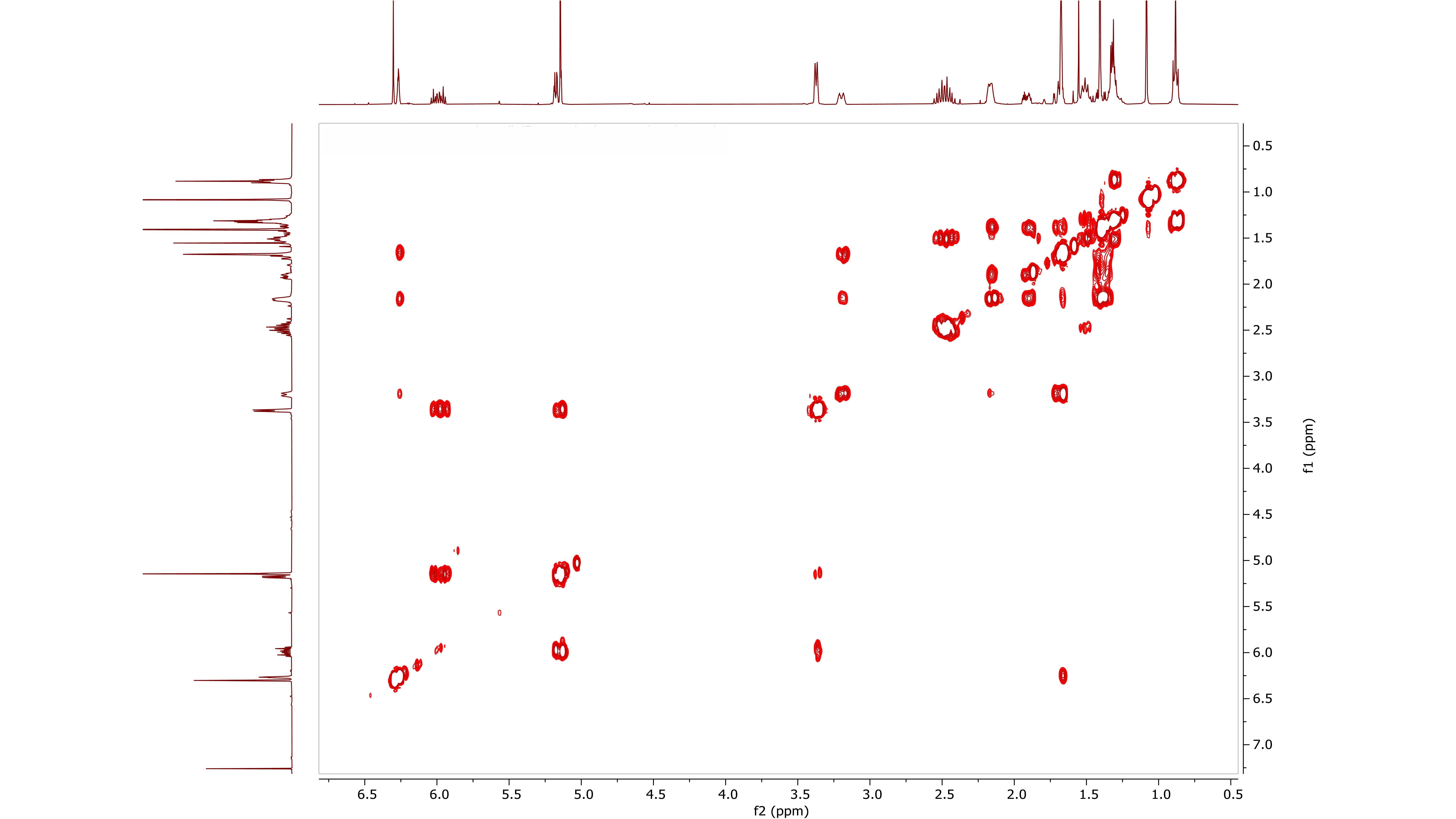


Figure S24: ^1^H- ^1^H COSY NMR spectra of "[2-propen-2-yl]-Δ^9^-tetrahydrocannabinol", in CDCl_3_.

Figure S25: Structure of "[2-propen-2-yl]-Δ^9^-tetrahydrocannabinol".

**“CBx” GC-MS and NMR analysis**

Figure S26: GC-MS chromatogram of "CBx" mixture (1).

Figure S27: GC-MS chromatogram of "CBx" mixture (2).

Figure S28: EI mass spectra of “[2-propen-2-yl]-Δ^8^-tetrahydrocannabinol”, [M]^+^= 354.2.


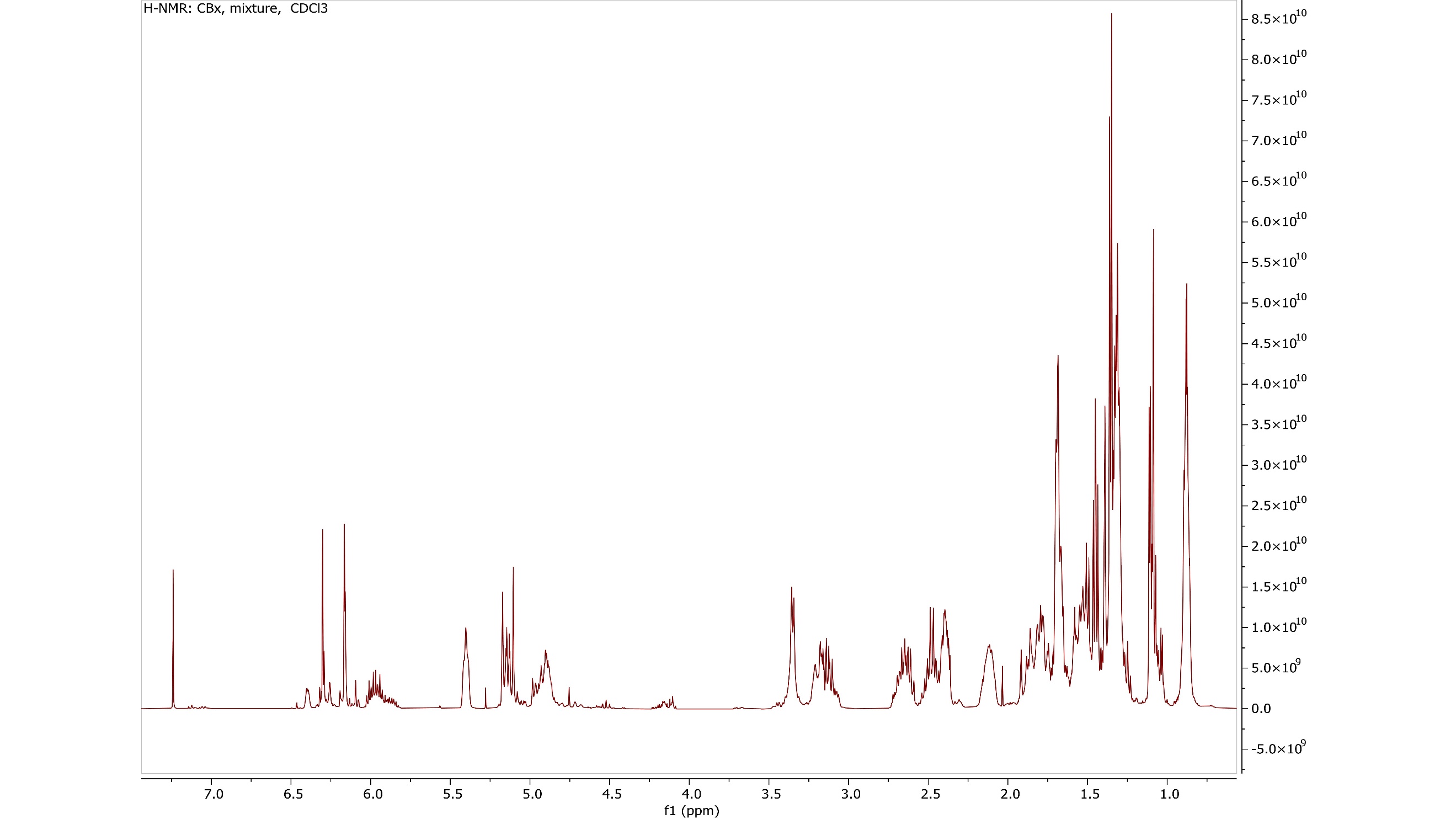


Figure S29: ^1^H NMR spectra of "CBx", in CDCl_3_, 400 MHz.

**
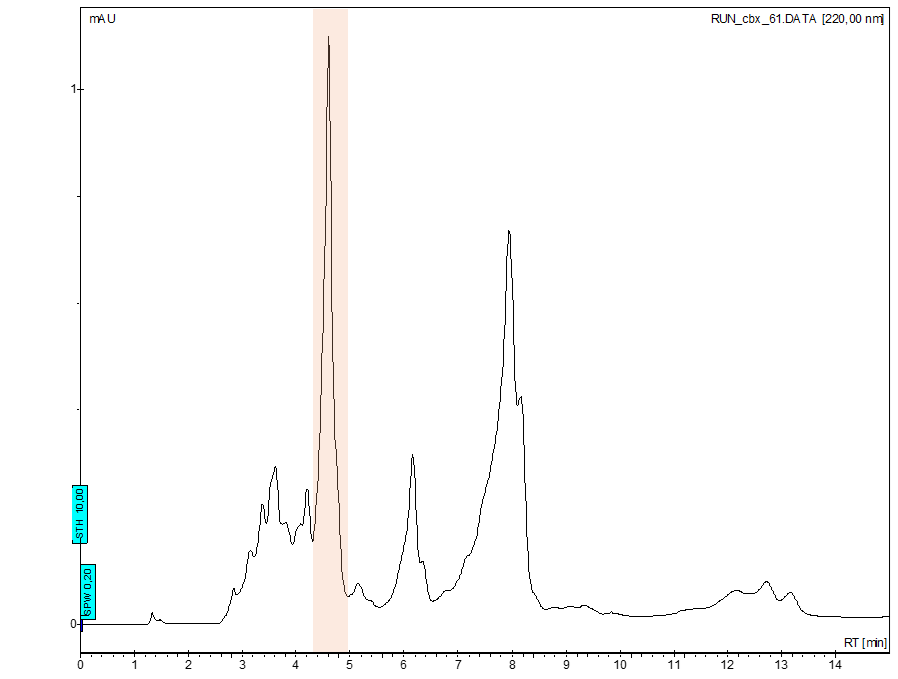
**

Figure 30: Preparative HPLC trace for the isolation of the major peak from the “CBx” mixture.


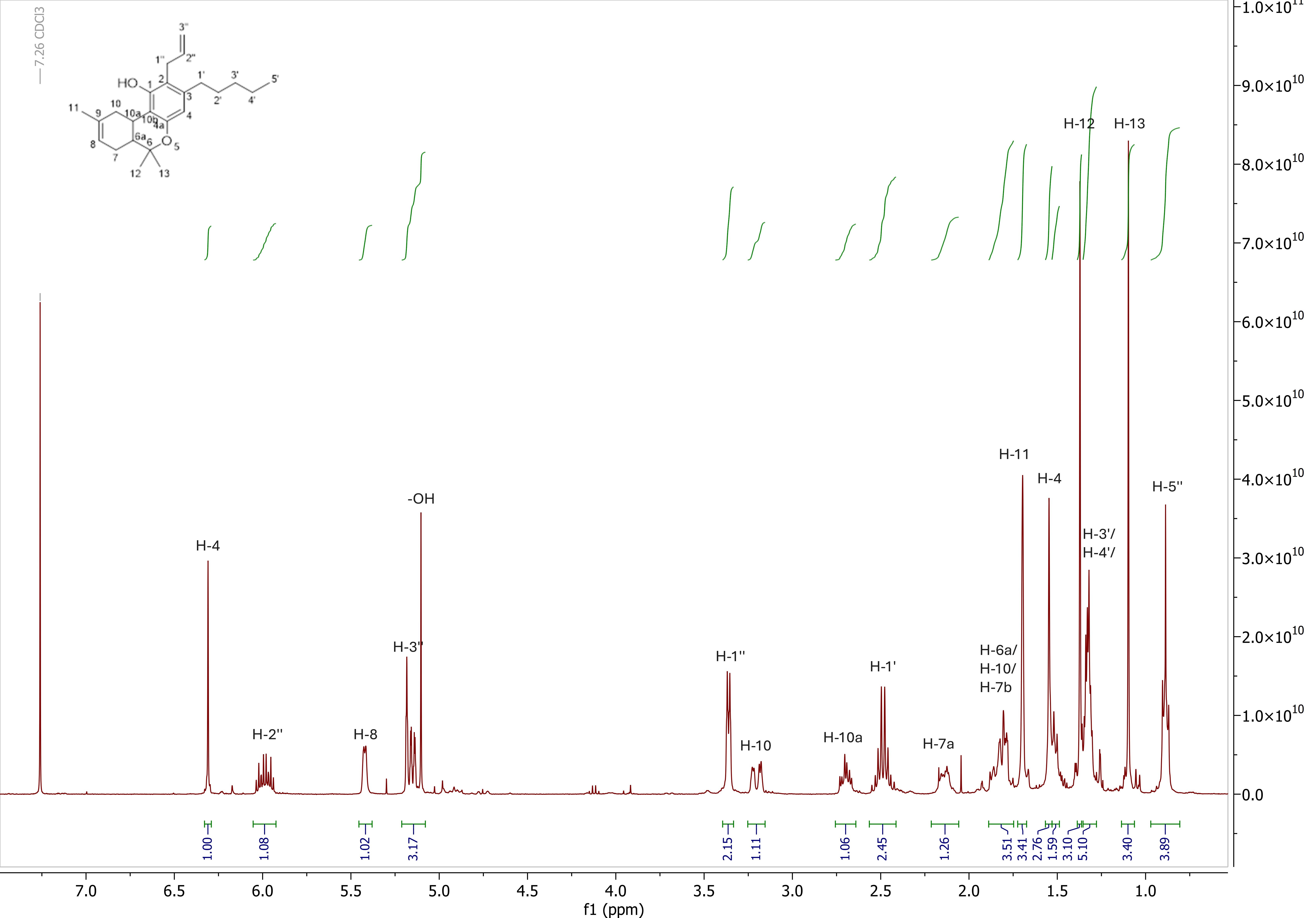


Figure S31: ^1^H NMR spectra of "[2-propen-2-yl]-Δ^8^-tetrahydrocannabinol", in CDCl_3_, 400 MHz.


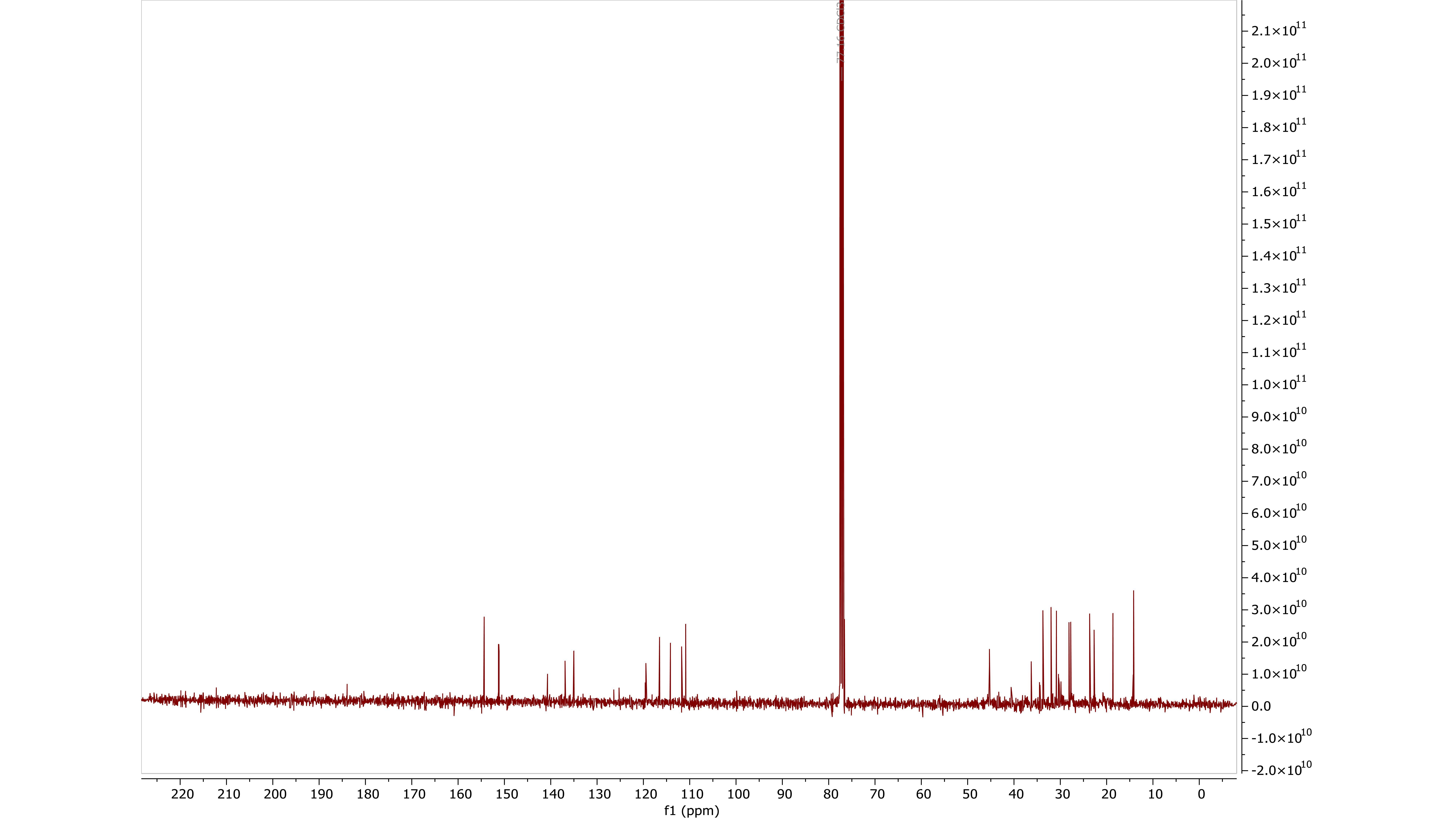


Figure S32: ^13^C NMR spectra of "[2-propen-2-yl]-Δ^8^-tetrahydrocannabinol", in CDCl_3_, 100 MHz.


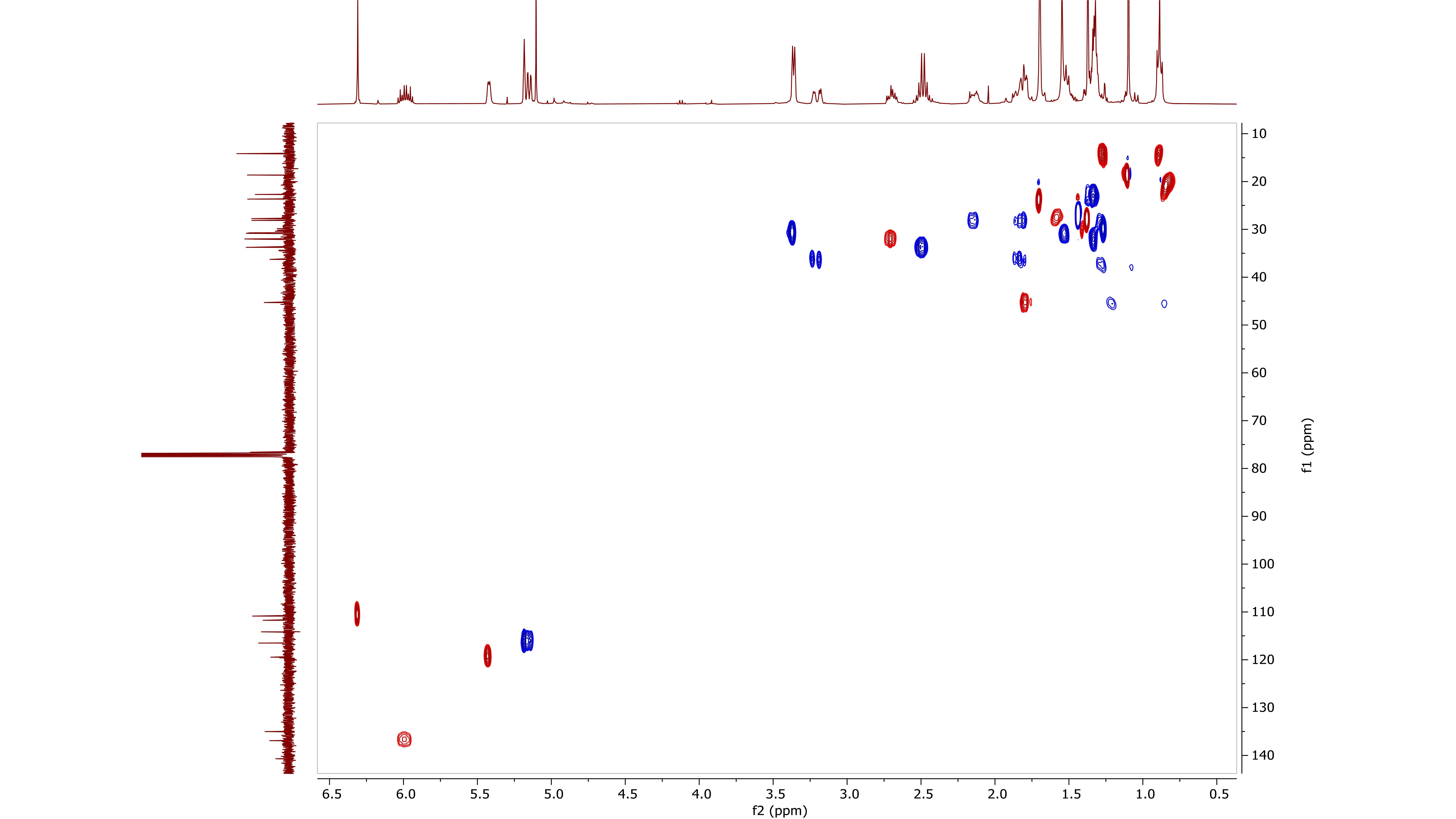


Figure S33: ^1^H- ^13^C HMQC-DEPT NMR spectra of "[2-propen-2-yl]-Δ^8^-tetrahydrocannabinol", in CDCl_3_.


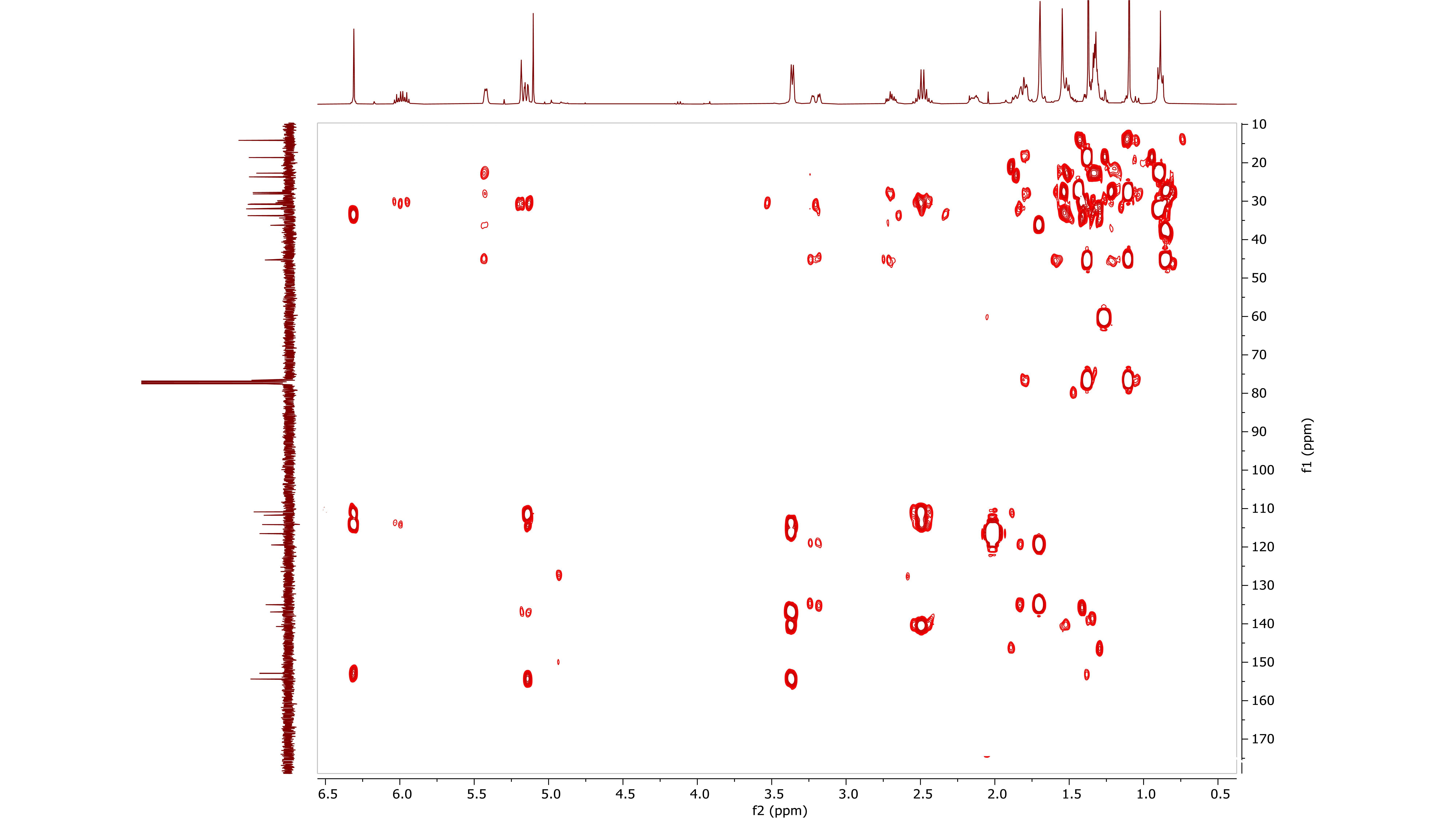


Figure S34: ^1^H- ^13^C HMBC NMR spectra of "[2-propen-2-yl]-Δ^8^-tetrahydrocannabinol", in CDCl_3_.

Figure S35: Structure of "[2-propen-2-yl]-Δ^8^-tetrahydrocannabinol".

Figure S36: Schematic representation of "[2-propen-2-yl]-Δ^8^-tetrahydrocannabinol" synthesis based on the patent EP0279308A2.
